# *SiLNR1*-Mediated Nitrogen Regulatory Signaling Enhances Nitrogen Use Efficiency and Grain Yield in Foxtail Millet (*Setaria italica* L.) under Low-Nitrogen Stress

**DOI:** 10.34133/research.1148

**Published:** 2026-02-25

**Authors:** Na Qin, Junxia Li, Senjie Fu, Cancan Zhu, Shutao Dai, Ya Jing, Chunyi Wang, Xin Wei, Chengyang Zhang, Zhenyan Ye, Yuhao Yuan, Xiaoqian Ma

**Affiliations:** ^1^ Cereal Crop Research Institute, Henan Academy of Agricultural Sciences, Zhengzhou 450002, China.; ^2^ School of Agricultural Sciences, Zhengzhou University, Zhengzhou 450001, China.; ^3^ College of Agronomy, Henan Agricultural University, Zhengzhou 450046, China.; ^4^ College of Agriculture, Henan University of Science and Technology, Luoyang 471000, China.

## Abstract

Foxtail millet (*Setaria italica* L.), a C4 cereal crop domesticated in China, exhibits exceptional nitrogen use efficiency (NUtE) with marked genotypic variation; however, the molecular basis remains underexplored. Here, we identified candidate genes through a sequential filtering strategy from genetic linkage (bulked segregant analysis sequencing) to transcriptional response (RNA sequencing) within defined quantitative trait locus intervals to dissect NUtE mechanisms in contrasting genotypes: the low-nitrogen-tolerant variety Yugu28 and the low-nitrogen-sensitive variety Qiyehuang, to identify the genetic regulatory mechanisms. Several candidate genes were screened, such as glutamine synthetase (*GS2*, Seita.3G024100), glutathione *S*-transferase (*GLUS*, Seita.3G386000) and nitrogen regulatory protein P-II (Seita.3G051900). Seita.3G051900 was identified as LOW-NITROGEN REGULATORY GENE (*SiLNR1*), which is highly expressed in different organs. The functional characterization revealed that *SiLNR1* overexpression results in strikingly low-N resilience: Compared with the wild-type controls, the transgenic lines presented 106% longer primary roots, 91.3% greater plant height, and 18.9% greater root nitrogen accumulation under low-nitrogen (LN) conditions and boosted grain yield and NUtE by 29.5% and 24.3%, respectively. By contrast, the *silnr1* mutant presented markedly shorter primary roots and plant height and lower shoot N accumulation under LN conditions. Field validation of the *SiLNR1* allele in the recombinant inbred line population demonstrated that the *SiLNR1* allele from Yugu28 confers a substantial and measurable yield gain under LN field conditions. The functions of *SiLNR1* associated with enhanced nitrogen uptake and utilization have been revealed, indicating that *SiLNR1* contributes to nitrogen metabolism and NUtE positively, thus providing a theoretical basis and application prospect for resource conservation, environmental protection, and sustainable agricultural development.

## Introduction

Nitrogen (N) is an essential macronutrient that fundamentally governs plant growth and developmental processes. The widespread use of nitrogen fertilizers has been instrumental in increasing crop productivity, serving as a key factor in ensuring consistently high yields. However, prolonged excessive N fertilization has diminished crop nitrogen use efficiency (NUtE), increasing agricultural production costs and triggering substantial environmental pollution. Thus, improving crop NUtE is imperative for sustainable agriculture [1,2].

Improving NUtE can be achieved through 2 complementary strategies: optimizing agronomic practices or enhancing inherent crop potential via molecular genetic approaches to modulate key genes governing nitrogen assimilation and utilization [3]. In arid and semiarid agricultural systems, nitrate (NO_3_^−^) serves as the predominant inorganic nitrogen source for crop uptake, a process governed by 4 evolutionarily conserved transporter families: NITRATE/PEPTIDE TRANSPORTER FAMILY (NPF), HIGH-AFFINITY NITRATE TRANSPORTER 2 (NITRATE), CHLORIDE CHANNEL (CLC), and *SLAC1*/*SLAH* (SLOW ANION CHANNEL-ASSOCIATED HOMOLOGS) [4]. On the basis of their NO_3_^−^ binding affinities, nitrate transporters are categorized into high-affinity transport systems, comprising *NRT2* proteins and their partner *Nar2* (NITRATE ASSIMILATION-RELATED 2), and low-affinity transport systems, which are primarily mediated by NRT1 proteins [5]. *NRT1.1* (NPF6.3) and *NLP7*, core transcriptional regulators of nitrate signaling, cooperatively mediate NO_3_^−^ signal perception and transduction in plants [6,7]. Recent advances in rice genomics have elucidated the biological functions of numerous NUtE-associated genes, particularly those encoding nitrate transporters that orchestrate NO_3_^−^ uptake and allocation. Notably, Hu et al. [8] demonstrated that allelic variation in *NRT1.1B* (NPF6.3) underlies the divergent NO_3_^−^ utilization capacities between Indica and Japonica subspecies, with *NRT1.1B* introgression markedly increasing NUtE in Japonica cultivars, thereby establishing this transporter as a prime target for precision breeding. Chen et al. [9] demonstrated that the transgenic overexpression of *OsNRT2.1*, driven by the *OsNAR2.1* promoter, synergistically enhanced nitrate (NO_3_^−^) acquisition, grain yield, and NUtE in rice. Notably, independent overexpression of *OsNAR2.1* alone similarly amplified NO_3_^−^ uptake capacity, leading to comparable improvements in both agronomic productivity and nitrogen-use efficiency [10], thereby validating the dual-target potential of this transporter-chaperone module for precision nitrogen management in cereal crops. In addition to nitrate transporters, transcription factors (TFs) are emerging as critical modulators of NUtE. The rice TF *OsNLP4* directly targets nitrate-responsive cis-elements to activate *OsNRT1.1B* (NPF6.3; nitrate uptake), *OsNRT2.4* (vascular loading), and *OsNIA1* (nitrate reductase; assimilation), thereby coordinating root-to-shoot nitrogen allocation. Genetic evidence has demonstrated that *OsNLP4* overexpression results in a 30% increase in grain yield and a 47% increase in NUtE [11,12], positioning this TF as a key regulator for breeding high-NUtE rice cultivars. Emerging evidence highlights that NUtE in rice is systemically coordinated via a transcriptional regulatory network involving multiple key factors: *MADS57* (MADS-box transcription factor 5), *NGR5* (nitrogen-mediated tiller growth response 5), *MYB61* (root nitrate foraging), *DREB1C* (drought-N crosstalk), *TBP2.1* (chromatin remodeling), *OsLBD37/38/39* (lateral root initiation), and *TCP19* (nitrogen-responsive photomorphogenesis). These TFs collectively fine-tune N allocation, assimilation, and remobilization through hierarchical control of downstream metabolic and developmental pathways [13–19].

As an important crop, foxtail millet (*Setaria italica* L.) is cultivated in arid and semiarid areas worldwide. Recent archeological evidence suggests that this species was domesticated ~11,000 years ago from its progenitor, green foxtail (*Setaria viridis*), in northern China [20]; it is characterized not only by high NUtE compared with other cereals but also by considerable variation in NUtE among its different genotypes [21]. Despite the importance of foxtail millet as a C4 model crop, the genetic architecture governing NUtE remains understudied. Genomic analyses revealed a minimalistic nitrogen transporter repertoire in foxtail millet, comprising 4 nitrate transporters (*NRTs*: *SiNRT1.1*, *SiNRT1.2*, *SiNRT2.1*, and *SiNRT2.4*), 2 ammonium transporters (*AMTs*: *SiAMT1.1* and *SiAMT1.3*), and 1 nitrate assimilation regulator (*SiNAR2.1*). The coordinated up-regulation of *SiNRT1.1*, *SiNRT2.1*, and *SiNAR2.1* synergizes with root architectural plasticity to increase nitrogen acquisition, as evidenced by a 1.8-fold increase in ^15^N influx rates [22]. Postabsorption, nitrate undergoes systemic redistribution via bidirectional translocation—root-to-shoot allocation through xylem loading and source-to-sink remobilization via phloem-mediated transfer from senescing leaves to developing tissue—a process fine-tuned by the spatial expression of these transporters [23–26]. Under prolonged extreme nitrogen limitation (0.02 mmol·l^−1^ NH_4_NO_3_ for 7 d), foxtail millet seedlings presented exceptional nitrogen economy through systemic nitrate remobilization, driven by 3.2-fold up-regulation of the vacuolar nitrate transporters *SiNRT1.11* and *SiNRT1.12* (NRT1/PTR family) in the shoot vasculature, facilitating source-to-sink redistribution of stored nitrate pools from senescing tissues to developing organs, whereas ammonium acquisition is concurrently fine-tuned by high-affinity ammonium transporters (*SiAMT1.1/1.3*) [27], demonstrating a dual-channel nitrogen scavenging strategy for survival under severe N deprivation. The ammonium transporter *SiAMT1.1* synergistically modulates enhanced nitrogen assimilation and optimized uptake strategies through transcriptional amplification under nitrogen-deprived conditions [22], positioning foxtail millet (*S. italica*) as a premier C4 model system for genetic dissection of NUtE [28,29]. Despite these advances, the regulatory circuitry governing genotype-dependent NUtE variation remains largely unknown. Our prior work identified Yugu28 (low-nitrogen-tolerant [LN-tolerant]) and Qiyehuang (low-nitrogen-sensitive [LN-sensitive]) as contrasting genotypes from a 35-accession panel, with pioneering studies employing multiomics approaches to decode their physiological divergence [30,31]. However, the core transcriptional dynamics and cis-regulatory logic underpinning their differential NUtE, particularly the hierarchical control of nitrate remobilization versus ammonium assimilation, demand systematic elucidation to establish foxtail millet as a blueprint for C4 nitrogen economization.

Bulked segregant analysis (BSA), a rapid and cost-efficient genomic strategy for mapping quantitative trait loci (QTLs), has emerged as a cornerstone in pinpointing phenotype-associated genes [32]. Here, we synergistically integrated BSA sequencing (BSA-seq) with transcriptome profiling (RNA sequencing [RNA-seq]) to dissect NUtE in foxtail millet [33,34], a C4 model crop. This multiomics framework narrowed candidate loci to a high-confidence subset governing nitrogen uptake and remobilization, including *SiLNR1* (LOW-NITROGEN REGULATORY 1), a nitrogen regulatory P-II protein homolog. Functional validation via overexpression assays in foxtail millet revealed that *SiLNR1* contributes to improve NUtE and plant growth, increasing plant architecture plasticity, grain yield, and nitrogen assimilation in *SiLNR1-OE* plants under low-nitrogen (LN) conditions. Our findings establish that *SiLNR1* is associated with enhanced NUtE, providing a mechanistic foundation for the biotechnological optimization of nitrogen economization in C4 cereals.

## Results

### Analysis of NUtE in the RIL population

**Fig. 1.**
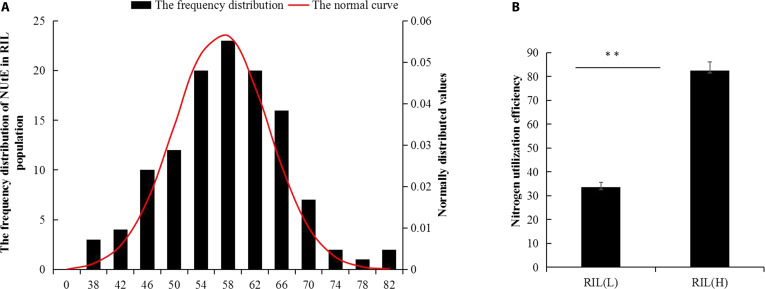
Evaluation of NUtE in a RIL population. (A) Distribution of NUtE phenotypes observed in the field. (B) Comparison of NUtE levels between the selected contrasting bulks. The values represent the means ± SEM (*n* = 15 biological replicates). The statistical significance of the difference was determined by Student *t* test (**P < 0.01). RIL(H), recombinant inbred line (high NUtE); RIL(L), recombinant inbred line (low NUtE).

After maturity, we used SPSS Statistics 26.0 to draw the NUtE frequency distribution map of 120 lines and carried out a normal distribution test. The results showed that skewness = −0.552 < 1 and kurtosis = 0.435 < 1, which indicated that the NUtE of 120 lines conformed to a normal distribution (Fig. 1A), so we speculated that the NUtE of millet in this population was a quantitative trait controlled by the main effect gene.

Leveraging the reduced genetic noise inherent to recombinant inbred line (RIL) populations, we performed BSA to resolve loci governing extreme-NUtE phenotypes. NUtE values between 25 and 35 g·g^−1^ are classified as low-NUtE types, whereas those with NUtE values greater than 75 g·g^−1^ are classified as high-NUtE types. Compared with the low-NUtE pool (RILL), the high-NUtE pool (RILH) presented 2.8-fold greater NUtE (Fig. 1B). This extreme phenotypic divergence provides exceptional statistical power for detecting allelic effects, validating our BSA-RIL integrative approach for dissecting complex traits in C4 crops.

### BSA-seq analysis

#### Evaluation of BSA-seq quality

Whole-genome resequencing of parental lines (Yugu28 and Qiyehuang) and extreme-NUtE pools [RIL(H)/RIL(L)] generated 46.97 Gb of raw sequencing data on the Illumina NovaSeq 6000 platform. Following adapter trimming and quality filtering (Trimmomatic v0.39; sliding window: 4 bp, mean phred ≥ 20), 44.80 Gb of high-confidence reads were retained (95.4% effective data rate). The following sequencing quality metrics met stringent standards: Q30 ≥ 90.63% (base call accuracy ≥ 99.9%), Q20 ≥ 96.07%, and GC content 46.61% to 49.81% (consistent with the *Setaria italica* v2.2 reference genome GC profile of 47.2%). The raw read error rates (0.03% to 0.05%) fell within Illumina’s specifications (<0.1%), validating the data fidelity for downstream variant analysis (Table S2).

#### Mapping analysis, detection, and annotation of SNPs

Read alignment to the *Setaria italica* reference genome (v2.2, Phytozome ID: GCA_000263155.2) yielded 302.1 million uniquely mapped reads, with alignment efficiencies of 98.71% to 99.22% across samples (Bowtie2 v2.4.5; end-to-end mode). The average whole-genome coverage was 23×, indicating a uniform read distribution (Kolmogorov–Smirnov test for coverage uniformity, *P* > 0.05; Table S3).

Variant calling via the Sentieon Genomics Suite (v202112.05) identified 2,072,744 high-confidence Single Nucleotide Polymorphisms (SNPs) after stringent filtering (QUAL ≥ 30, DP ≥ 10, GQ ≥ 20). Functional annotation via ANNOVAR categorized 57,798 mutations as nonsynonymous substitutions, 1,145 as stop-gain mutations, and 165 as stop-loss mutations (Table S4). Notably, 12.4% of exonic SNPs localized to conserved protein domains, suggesting potential functional impacts on nitrogen-associated pathways.

#### Locations of candidate regions and screening of genes

**Fig. 2.**
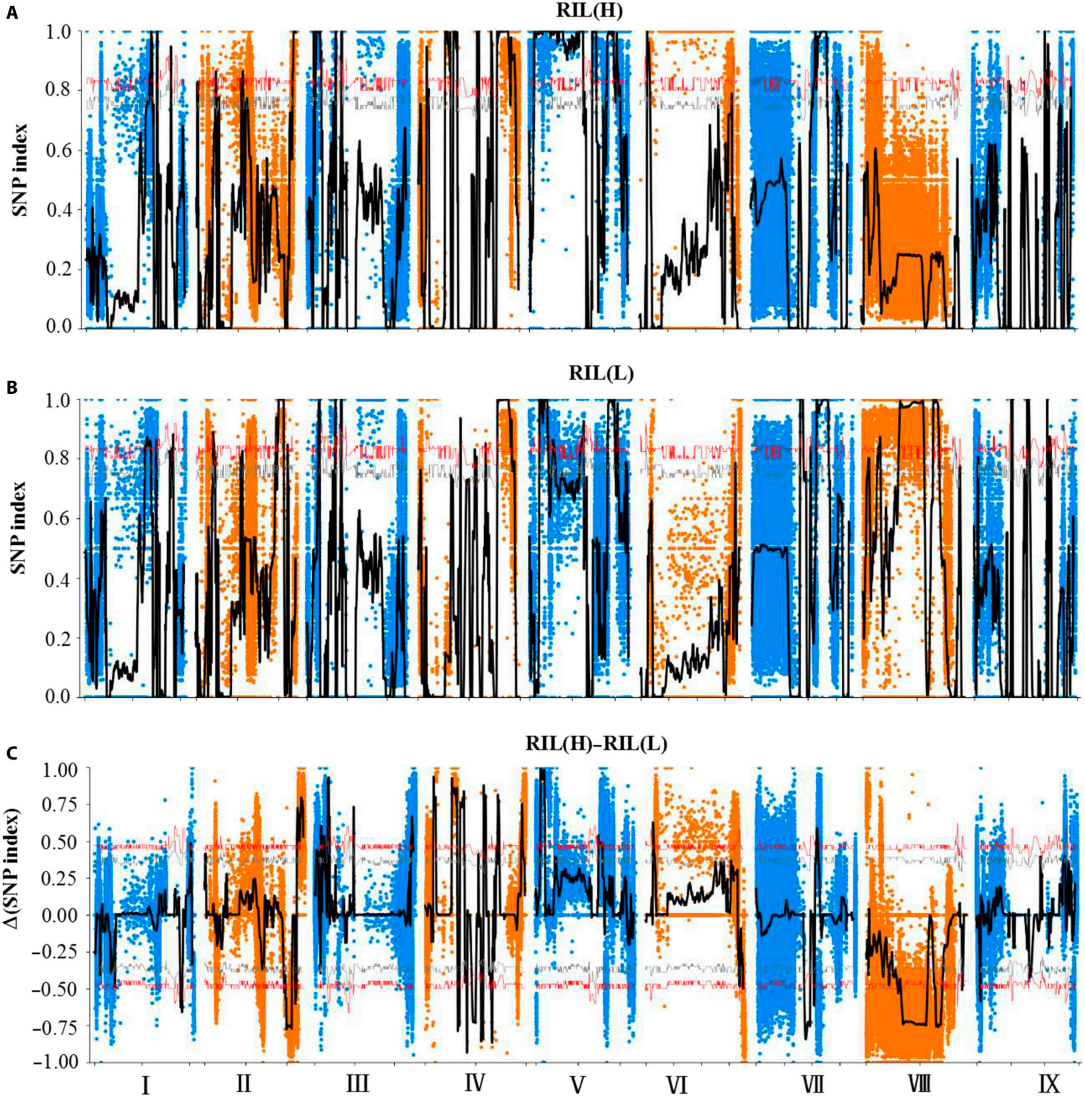
BSA-seq analysis for identifying genomic loci associated with NUtE variation. Manhattan plots displaying the distribution of SNP index values across the genome for the high-NUtE RILs (A), the low-NUtE RILs (B), and their difference (ΔSNP index) (C). The gray horizontal line in (C) indicates the 95% confidence interval, with genomic regions exceeding this threshold considered candidate intervals.

From 560,964 homozygous polymorphic markers, genome-wide SNP indices were computed for extreme-NUtE pools (RILH/RILL). The high correlation of SNP index values between replicate pools confirmed the consistency of our bulk construction (Fig. 2A and B). Delta (Δ) SNP indices were derived via a sliding window approach (1-Mb window, 100-kb step), revealing loci under divergent selection pressure. Permutation testing (*n* = 1,000 iterations) established a genome-wide significance threshold at the 95th percentile (ΔSNP index ≥ 0.5, *P* < 0.05; Bonferroni-corrected). As a result, a total of 3,913 loci were ultimately identified, including 28,306,291 to 48,857,382 bp of chromosome II; 850,628 to 50,618,069 bp of chromosome III; 4,239,431 to 39,660,439 bp of chromosome IV; 2,391,445 to 32,517,605 bp of chromosome V; 3,512,113 to 35,908,517 bp of chromosome VI; 21,855,880 to 23,356,484 bp of chromosome VII; 6,019,925 to 29,079,447 bp of chromosome VIII; and 2,020,541 to 52,253,458 bp of chromosome IX (Fig. 2C). We identified 668 nonsynonymous mutation sites within the exons of the candidate region, which are predicted to have functional consequences. These mutations were located in a total of 166 genes (Table S5).

### Transcriptional responses of LN-tolerant foxtail millet and LN-sensitive foxtail millet to the N supply at the seedling stage

To elucidate the molecular mechanism of the difference in NUtE between LN-tolerant and LN-sensitive foxtail millet, the expression profiles of the leaves of 2-parent materials (Yugu28 and Qiyehuang) at the seedling stage were identified under control (CK: 2.5 mmol·l^−1^ NO_3_^−^) and LN (0.5 mmol·l^−1^ NO_3_^−^) conditions. Principal component analysis revealed clear separation between genotypes and treatments, with biological replicates clustering tightly, indicating high data quality (Fig. S1).

High-confidence RNA-seq data were generated (mean, 6.23 Gb/sample; Q30 ≥ 93.80%, mapping rate, 95.54%; Table S6). DESeq2 analysis (false discovery rate [FDR] < 0.05, |log2FC| >1) revealed profound genotype-driven expression divergence. Differentially expressed genes (DEGs) analysis (FDR < 0.05, |log2FC| > 1) was performed by comparing 2 genotypes (CK_Yugu28 versus CK_Qiyehuang and LN_Yugu28 versus LN_Qiyehuang) and 2 N levels (CK_Yugu28 versus HN_Yugu28 and CK_Qiyehuang versus LN_Qiyehuang). There were 17,385 DEGs between Yugu28 and Qiyehuang genotypes under the CK level and 14,664 DEGs under the LN level (Fig. S2A). Owing to the distinct genetic backgrounds of the 2 parental genotypes, many genes were differentially expressed even under control conditions. The results showed that 1,189 DEGs were changed in response to N supply in the LN-tolerant genotype (Yugu28), and 653 DEGs were changed in response to N supply in the LN-sensitive genotype (Qiyehuang) (Fig. S2B).

Functional enrichment (Gene Ontology [GO]/Kyoto Encyclopedia of Genes and Genomes [KEGG]) highlighted LN-responsive pathways in Yugu28, including nitrogen assimilation (Glutamine Synthetase/Glutamate Synthase cycle; *P*adj = 1.2 × 10^−9^), root architecture modulation (auxin transport; *P*adj = 3.8 × 10^−6^), and C4 photosynthetic optimization (nicotinamide adenine dinucleotide phosphate-dependent malic enzyme; *P*adj = 7.4 × 10^−5^). Quantitative real-time polymerase chain reaction (qRT-PCR) validation of the 9 prioritized DEGs strongly agreed with the RNA-seq profiles (Fig. S3), confirming the reliability of the dataset.

**Fig. 3.**
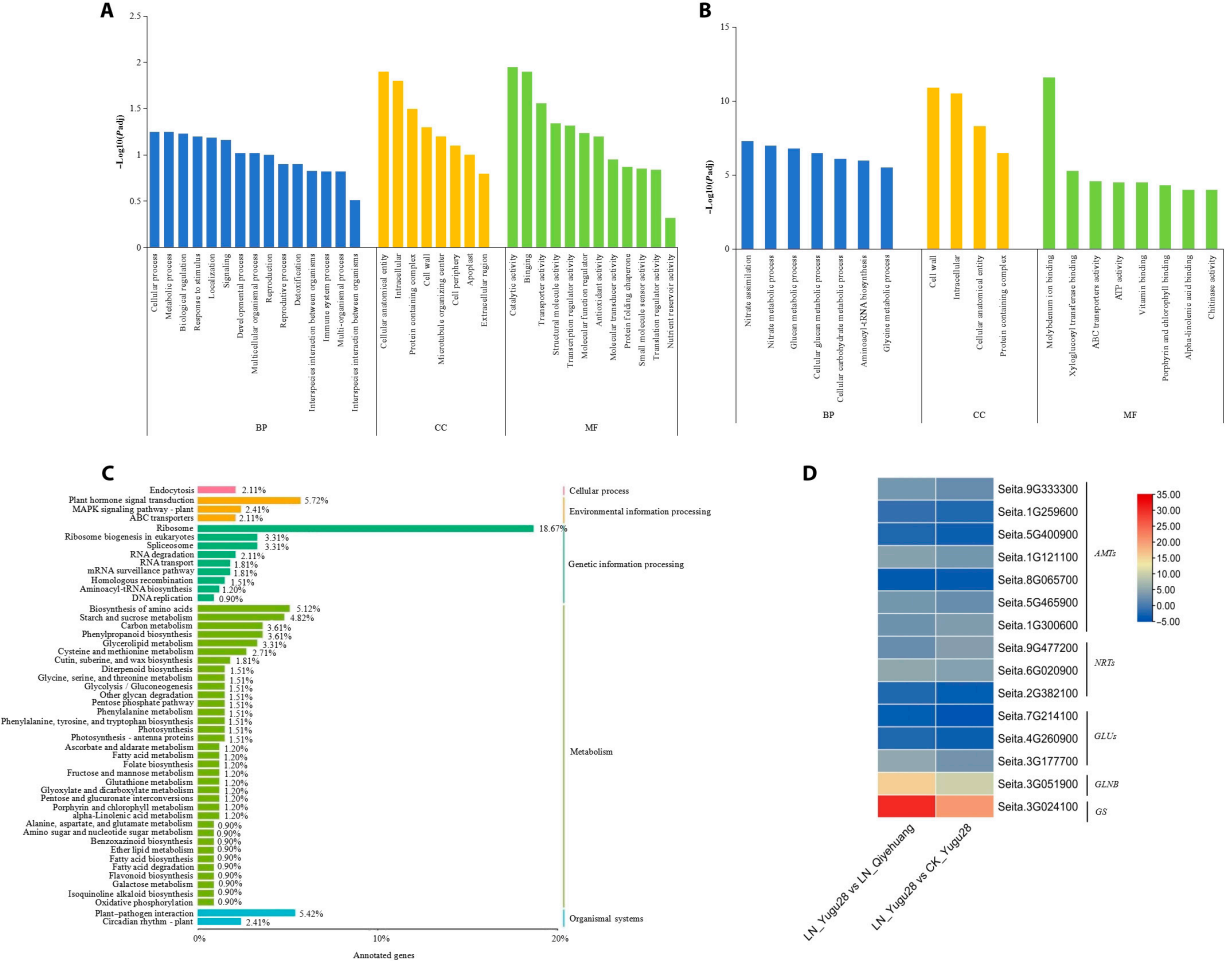
Multilevel enrichment mapping of LN-responsive DEGs between Yugu 28 and Qiyehuang. GO and KEGG analyses of DEGs in the leaves of the 2 foxtail millet genotypes under LN conditions (A and C). GO analysis of DEGs in the leaves of the LN-tolerant genotype between high-nitrogen and LN conditions (B). Heatmap of the expression patterns of DEGs enriched in the N metabolic pathway (D). “BP”, “CC”, and “MF” indicate “biological process”, “cellular component”, and “molecular function”, respectively.

Functional enrichment analysis of DEGs under LN conditions revealed distinct response patterns between genotypes. The GO terms associated with the common DEGs in both genotypes were associated with cellular processes and regulatory activity (Fig. 3A), suggesting a role in nutrient sensing. In contrast, LN tolerance-specific DEGs were uniquely enriched in nitrogen metabolism and assimilation pathways (Fig. 3B). KEGG analysis further highlighted shared enrichment in core metabolic processes, including amino acid and carbon metabolism, plant hormone signaling, and phenylpropanoid biosynthesis. Notably, the nitrogen metabolism pathway included key genes, such as *AMTs*, *NRTs*, *GSs*, *GLNBs*, and *GLUs*, that directly link transcriptional regulation to NUtE (Fig. 3C).

The expression levels of Seita.3G051900 (nitrogen regulatory protein P-II, *GLNB*) and Seita.3G024100 (glutamine synthetase [*GS*]) in LN-tolerant foxtail millet were approximately 30 and 15 times greater than those in LN-sensitive foxtail millet under LN conditions (Fig. 3D), respectively, which may play essential roles in regulating N metabolism and NUtE.

### Identification of NUtE candidate genes via combined BSA-seq with RNA-seq

To further prioritize the candidate genes of NUtE in foxtail millet, we analyzed the differential expression of 166 genes with nonsynonymous mutations identified by BSA-seq between the high-NUtE and low-NUtE genotypes. The results revealed that 6 core genes underpin NUtE divergence between LN-tolerant (Yugu28) and LN-sensitive (Qiyehuang) genotypes under LN conditions. The corresponding functional annotations indicate that these genes encode glutamine synthetase, nitrogen regulatory protein P-II, glutathione *S*-transferase, wall-associated receptor kinase, HEAT repeat family protein, and protein-l-isoaspartate *O*-methyltransferase (Table 1).

**Table 1.** Description of candidate genes

Gene ID	Homologous gene in rice	Expression patterns of LN_Yugu28 VS LN_Qiyehuang (fold change)	Functional annotation
Seita.3G024100	Os04g56400.1	15.01	Glutamine synthetase
Seita.3G051900	Os05g04220.1	30.36	Nitrogen regulatory protein P-II
Seita.3G386000	Os09g37240.1	−4.30	Glutathione *S*-transferase, C-terminal
Seita.3G392800	Os12g42070.1	4.10	Wall-associated receptor kinase 2
Seita.6G048000	Os08g07290.1	−3.22	HEAT repeat family protein
Seita.7G140000	Os04g40540.1	−3.80	Protein-l-isoaspartate *O*-methyltransferase

**Fig. 4.**
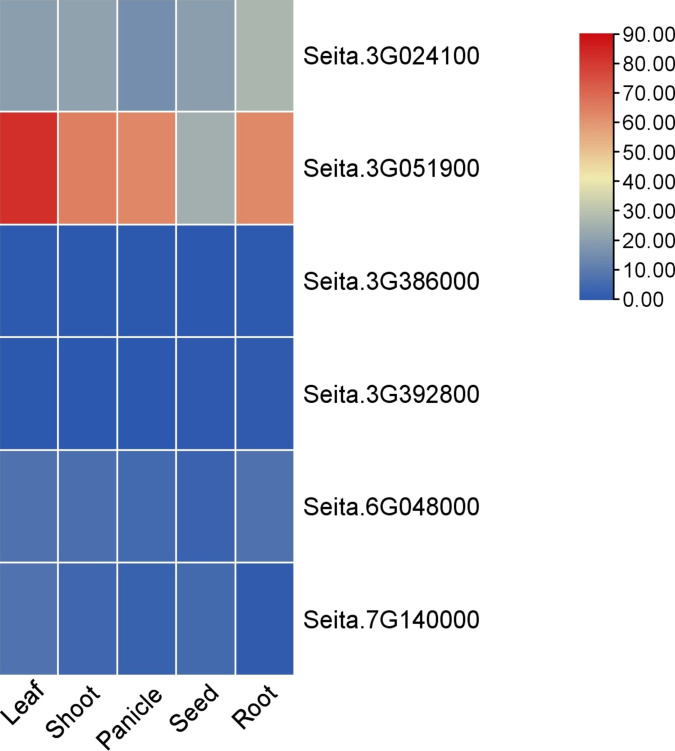
Spatial expression profiles of candidate NUtE-associated genes. The heatmap displays the transcript abundance (log2 fragments per kilobase of transcript per million) of the candidate genes across 5 distinct tissues, revealing constitutive or tissue-preferential expression patterns.

Among them, the differential expression ratios of Seita.3G024100, Seita.3G051900, Seita.3G392800, and Seita.3G386000 were greater (fold change >4) (Table 1). The BSA-seq interval Seita.3G051900 showed substantial overlap with a major-effect QTL for NUtE that we had previously mapped in the same RIL population [31]. Expression profiling across 5 organ types revealed that the gene encoding the nitrogen regulatory P-II protein (*GLNB*, Seita.3G051900) presented high constitutive expression in leaves, shoots, panicles, and roots (Fig. 4). The candidate gene Seita.3G051900 was named *SiLNR1* (LN regulatory gene). Four amino acid deletions in the *SiLNR1* protein sequence between LN-tolerant variety Yugu28 and LN-sensitive variety Qiyehuang occurred outside the conserved domain, and two of these were predicted to be phosphorylated sites. In Yugu28, there was an increase in Ser67, and in Qiyehuang, alanine (Ala59) was transformed into Ser59 (Fig. S4). Owing to its uncharacterized role in NUtE regulation, *SiLNR1* (Seita.3G051900) was selected as the primary candidate for functional investigation. Phylogenetic analysis revealed that *SiLNR1* is highly conserved in grasses, with the highest amino acid sequence identity with putative orthologs in *Panicum virgatum* (switchgrass, 87%) and *Sorghum bicolor* (sorghum, 83%). This high degree of sequence conservation, particularly within the Panicoideae subfamily, suggests that the molecular function of LNR1-like proteins may be conserved (Fig. S5).

### *SiLNR1* promotes root elongation and plant height and enhances NUtE under LN conditions

**Fig. 5.**
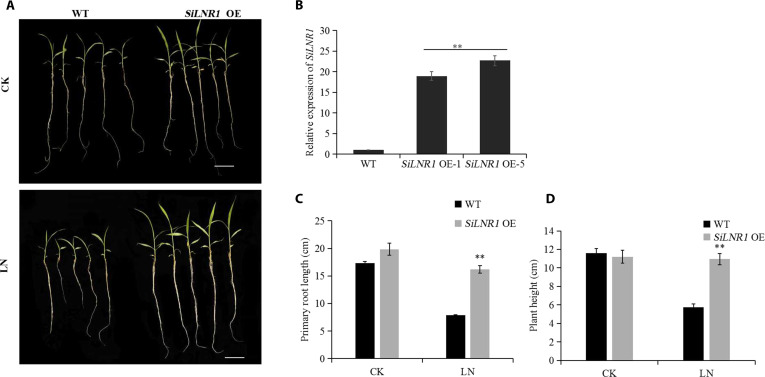
Phenotypic identification of *SiLNR1*-overexpressing plants. Phenotypes of the WT and *SiLNR1*-overexpressing plants under CK and LN conditions (A), relative expression of *SiLNR1* in foxtail millet determined via qRT-PCR (B), primary root length (C), and plant height (D). The data are presented as the means ± SD (*n* = 6 biologically independent plants). Asterisks indicate statistically significant differences (*P < 0.05, **P < 0.01) according to Student *t* test. CK, control (2.5 mmol·l^−1^ NO_3_^−^); LN, low-nitrogen stress (0.5 mmol·l^−1^ NO_3_^−^); *SiLNR1* OE, *SiLNR1*-overexpressing.

**Fig. 6.**
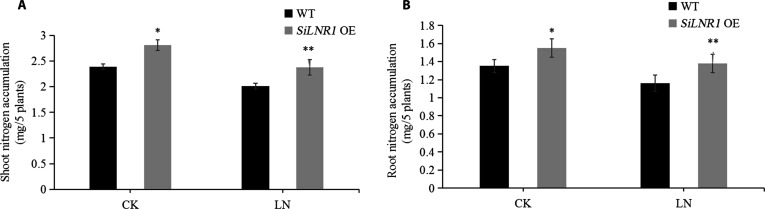
N accumulation in the shoots and roots of *SiLNR1*-overexpressing transgenic plants. The bars indicate the means ± SEs (*n* = 6). Asterisks indicate statistically significant differences (*P < 0.05, **P < 0.01) according to Student *t* test. CK, control (2.5 mmol·l^−1^ NO_3_^−^); LN, low-nitrogen stress (0.5 mmol·l^−1^ NO_3_^−^); *SiLNR1* OE, *SiLNR1*-overexpressing.

We overexpressed *SiLNR1* in foxtail millet and used the identified homozygous seeds for phenotypic identification on 1/2-strength nitrogen-free Murashige and Skoog medium with different NO_3_^−^ concentrations. At the LN (0.5 mmol·l^−1^ NO_3_^−^) level, *SiLNR1-OE* plants presented dramatic phenotypic superiority: The primary roots were 106% longer (16.20 ± 1.1 cm versus 7.86 ± 0.7 cm, respectively; *P* < 0.01, Student *t* test), and the plant height was 91.3% greater than that of the wild type (WT) (10.94 ± 0.6 cm versus 5.72 ± 0.4 cm, respectively; *P* < 0.01, Student *t* test) (Fig. 5). Compared with the WT plants, the *SiLNR1-OE* plants presented 18.9% greater root nitrogen accumulation (1.38 ± 0.09 mg/5 plants versus 1.16 ± 0.07 mg/5 plants; *P* < 0.01, Student *t* test), whereas the shoot nitrogen accumulation was elevated by 18.4% under the LN treatment (2.38 ± 0.10 mg/5 plants versus 2.01 ± 0.05 mg/5 plants; *P* < 0.01, Student *t* test) and 17.5% under the control conditions (CK: 2.5 mmol·l^−1^ NO_3_^−^) (Fig. 6A and B).

Analysis of multiple independent *SiLNR1-OE* lines revealed that, compared with the WT plants, the *SiLNR1-OE* lines consistently presented significant increases in plant height, root length, and nitrogen accumulation under both control and LN conditions (Fig. S6).

**Fig. 7.**
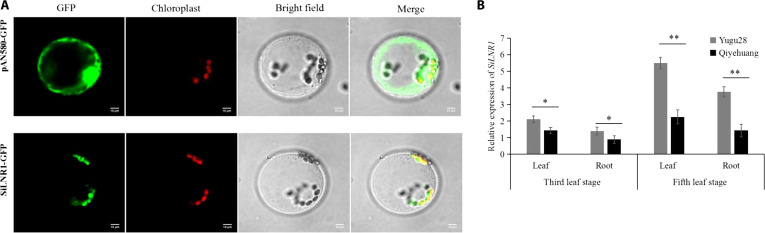
Expression pattern of *SiLNR1*. Subcellular localization of *SiLNR1* (A). Tissue-specific expression of *SiLNR1* (B). The bars indicate the means ± SEs (*n* = 3). Asterisks indicate statistically significant differences (*P < 0.05, **P < 0.01) according to Student *t* test.

These findings suggest that *SiLNR1* is likely involved in the N response. Subcellular localization revealed that *SiLNR1* was a regulatory gene localized to the chloroplast (Fig. 7A). qRT-PCR revealed that the expression of *SiLNR1* in Yugu28, which is expressed mainly in leaves at the seedling stage, was greater than that in Qiyehuang (Fig. 7B).

**Fig. 8.**
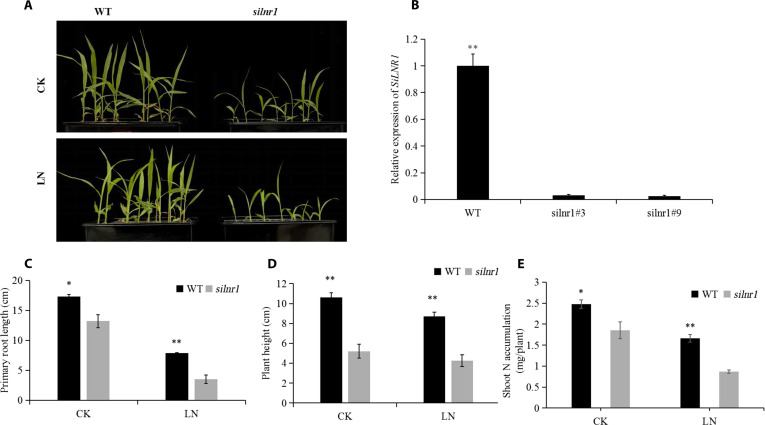
Phenotypic identification of *silnr1* mutants. Phenotypes of the WT and *silnr1* mutants under CK and LN conditions (A), relative expression of *SiLNR1* in foxtail millet determined via qRT-PCR (B), primary root length (C), plant height (D), and shoot N accumulation (E). The bars indicate the means ± SEs (*n* = 6). Asterisks indicate statistically significant differences (*P < 0.05, **P < 0.01) according to Student *t* test. CK, control (2.5 mmol·l^−1^ NO_3_^−^); LN, low-nitrogen stress (0.5 mmol·l^−1^ NO_3_^−^); *silnr1*, *silnr1* mutant.

Compared with the WT, the *silnr1* mutant of foxtail millet presented importantly shorter primary roots and plant heights and lower shoot N accumulation under CK and LN conditions (Fig. 8). We also generated 3 independent *silnr1* mutant lines. Phenotypic evaluation under LN stress revealed that all the mutant lines exhibited increased sensitivity compared with the WT. The consistent phenotypic data for all independent lines are provided in Fig. S7.

**Fig. 9.**
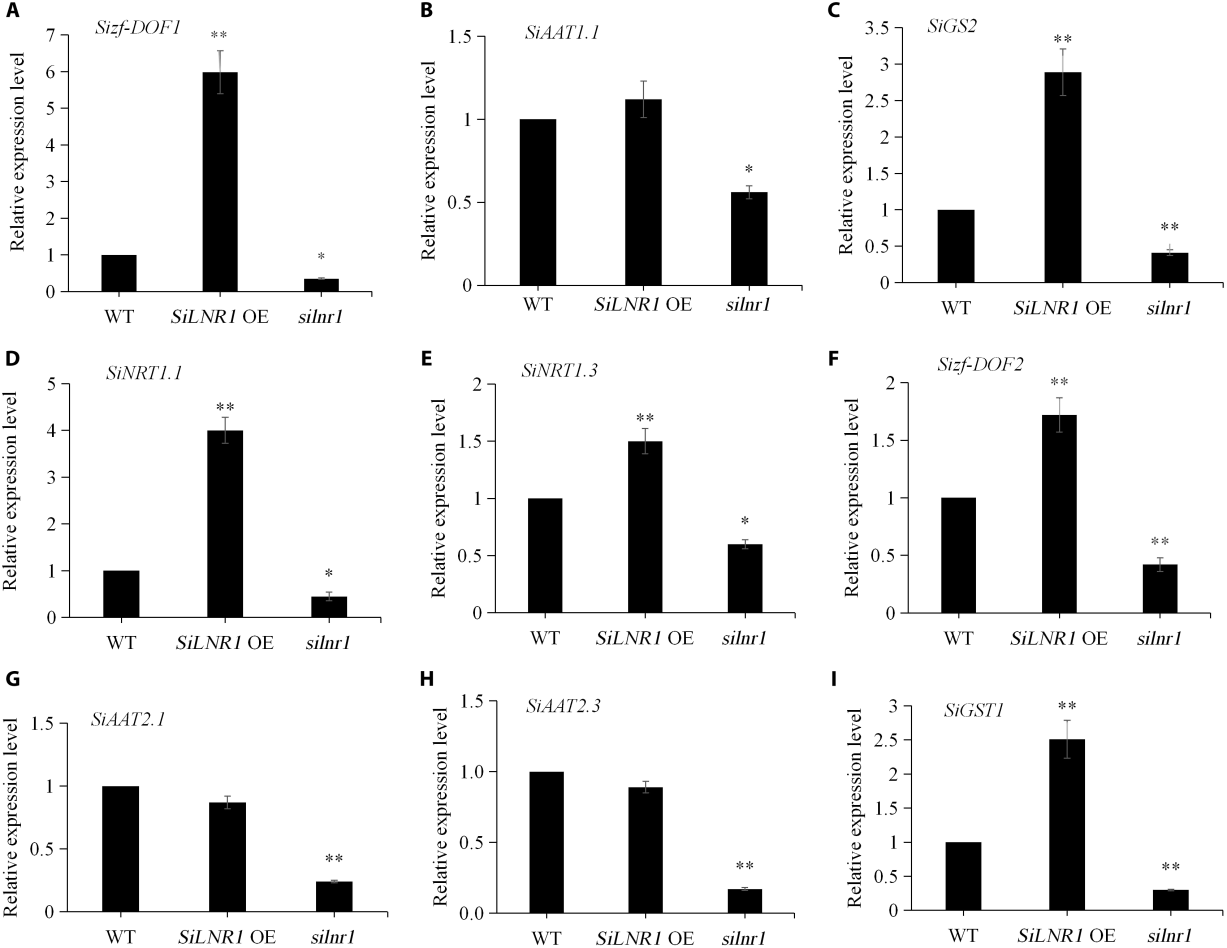
Expression analysis of genes related to N metabolism, N assimilation, the N response and N transport in *SiLNR1-*overexpressing plants and *silnr1* mutants. Relative expression levels of genes *Sizf-DOF1* (A), *SiAAT1.1* (B), *SiGS2* (C), *SiNRT1.1* (D), *SiNRT1.3* (E), *Sizf-DOF2* (F), *SiAAT2.1* (G), *SiAAT2.3* (H), and *SiGST1* (I). Three-week-old seedlings of WT, *SiLNR1*-overexpressing plants and *silnr1* mutants grown in a nutrient mixture supplemented with 2.5 mM NO_3_^−^ were sampled for gene expression analysis. The bars indicate the means ± SEs (*n* = 3). Asterisks indicate statistically significant differences (*P < 0.05, **P < 0.01) according to Student *t* test. *SiLNR1* OE, *SiLNR1*-overexpressing; *silnr1*, *silnr1* mutant.

The expression levels of genes associated with N metabolism (protein NRT, *NRT1.1* and *NRT1.3*), N assimilation (glutamine synthetase 2 [*GS2*], glutathione *S*-transferase 1, and *GST1*), and N response factors (Dof zinc finger protein, *zf-Dof1* and *zf-Dof2*) were increased in the *SiLNR1*-overexpressing plants and were 4.0-, 1.5-, 2.9-, 2.5-, 6.0-, and 1.7-fold greater than those in the WT plants, respectively. The expression levels of other genes related to N transport (amino acid transporter, *AAT1.1*, *AAT2.1*, and *AAT2.3*) were similar between the *SiLNR1*-overexpressing and WT plants (fold change <1.5), while the expression of these genes were all reduced significantly in the *silnr1* mutants (Fig. 9).

**Fig. 10.**
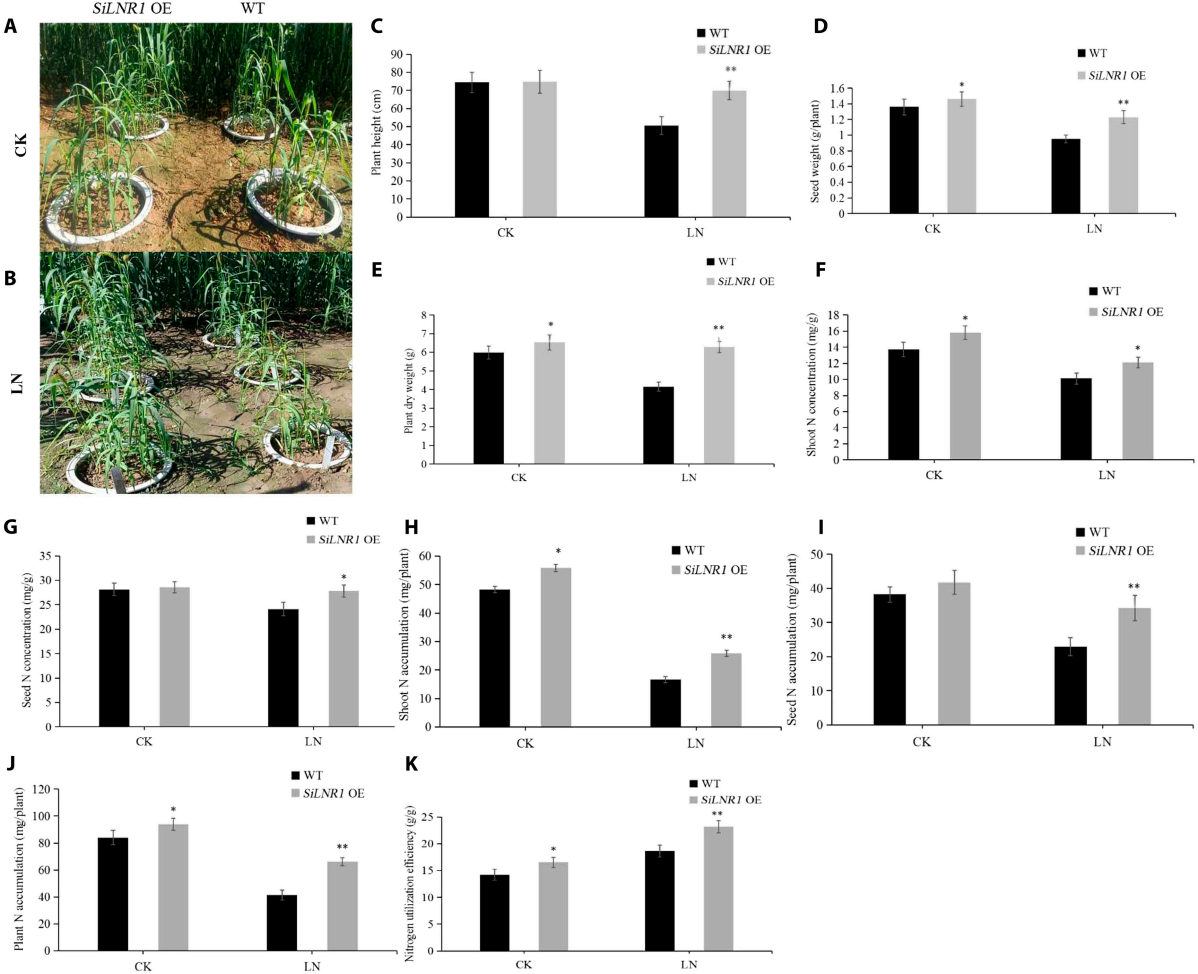
Phenotypes and related indices of *SiLNR1*-overexpressing plants at the maturity stage. Phenotypes of WT and *SiLNR1*-overexpressing (*SiLNR1* OE) foxtail millet plants under CK (A) and LN (B) conditions at the mature stage. Plant height, seed weight, and dry weight of WT and *SiLNR1*-overexpressing plants (C to E). Shoot (F) and seed (G) N concentrations, shoot (H) and seed (I) N accumulation, plant N accumulation (J), and NUtE (K) of WT and *SiLNR1*-overexpressing plants. The bars indicate the means ± SEs (*n* = 6). Asterisks indicate statistically significant differences according to Student *t* test (^*^P < 0.05, ^**^P < 0.01). CK, control (2.5 mmol·l^−1^ NO_3_^−^); LN, low-nitrogen stress (0.5 mmol·l^−1^ NO_3_^−^).

The results of the pot experiment revealed that *SiLNR1-OE* plants presented 38.4% greater plant height, 29.5% greater seed weight per plant, and 19.8% greater shoot nitrogen concentration, and NUtE increased by 24.3% compared with those of the WT controls (Fig. 10C, D, F, and K) under LN conditions. Compared with WT plants, *SiLNR1-OE* plants maintained agronomic parity under CK conditions, with no significant differences in terms of plant height, seed N concentration, or seed N accumulation (Fig. 10C, G, and I). The improved nitrogen uptake and metabolism in *SiLNR1-OE* plants were specific to the LN condition. Thus, our genetic evidence supports the idea that *SiLNR1* provides a functional advantage in coordinating nitrogen economy under nutrient stress.

### Field validation of the *SiLNR1* allele in the RIL population

To evaluate the agronomic impact of the natural *SiLNR1* variation, we performed a field trial under LN conditions using the RIL population. Based on genotyping, 15 RILs carrying the LN-tolerant allele (Yugu28) and 15 carrying the LN-sensitive allele (Qiyehuang), along with both parents, were selected for detailed agronomic traits.

The RILs with the tolerant *SiLNR1* allele exhibited significantly superior performance across key agronomic traits under LN stress. Specifically, compared to the sensitive allele group, the tolerant group displayed 10.9% greater plant height (*P* < 0.05), 26.1% higher grain yield per plant (*P* < 0.01), and a 16.8% increase in thousand grain weight (*P* < 0.05) (Table 2). Critically, this yield advantage was underpinned by a concomitant 24.9% improvement in NUtE (*P* < 0.01), directly linking the allele to enhanced nitrogen utilization efficiency in the field. The performance of individual RILs spanned the range between the 2 parents, confirming the genetic segregation of this locus.

**Table 2.** Yield trait analysis of the RIL population and its parents under LN conditions. The bars indicate the means ± SEs (*n* = 15). Asterisks indicate statistically significant differences according to Student *t* test (^*^P < 0.05, ^**^P < 0.01).

Traits	Parents		*t* test	RIL populations		*t* test
	Yugu28	Qiyehuang		LN-tolerant allele	LN-sensitive allele	
Plant height (cm)	120.70 ± 4.22	103.13 ± 3.15	**	120.02 ± 3.21	108.51 ± 2.14	*
Grain yield per plant (g)	19.53 ± 0.89	14.34 ± 0.63	**	18.97 ± 0.97	15.04 ± 0.84	**
Thousand grain weight (g)	2.82 ± 0.08	2.25 ± 0.06	**	2.71 ± 0.02	2.32 ± 0.04	*
NUtE (g/g)	21.32 ± 1.02	16.50 ± 1.14	**	20.89 ± 1.14	16.73 ± 0.95	**

## Discussion

### Coordinated expression of N metabolism genes toward an understanding of NUtE

Nitrogen metabolism and assimilation are central to optimizing NUtE and crop productivity [35]. Our KEGG analysis revealed pronounced enrichment of nitrogen metabolic pathways in the LN-tolerant genotype Yugu28, driven by coordinated up-regulation of key enzymes—*AMTs*, *NRTs*, *GSs*, *NRs*, and *GLUs* (Fig. 3C). Strikingly, *GS2* (Seita.3G024100), encoding a glutamine synthetase, exhibited 15-fold higher expression in Yugu28 than in the LN-sensitive Qiyehuang (Table 1). Consistent with our findings, its expression is elevated in LN-tolerant soybean genotypes under nitrogen stress [36]. Furthermore, functional studies in *Arabidopsis* have demonstrated that *GS2* is indispensable for nitrogen uptake, with mutants exhibiting a severe (70%) reduction in uptake activity [37], and its role in rice has also been well established. Shambhu et al. [38] reported that the overexpression of rice *GS2* genes increases the expression of photosynthesis-related enzymes and chlorophyll pigment accumulation, ultimately conferring a 31% to 40% improvement in NUtE in transgenic rice plants. Mondal et al. [39] performed a comprehensive investigation of the stage-specific expression pattern of GS2 transcripts and reported that *ATGS2* was more highly expressed in *Arabidopsis* and that *OsGS2* was more highly expressed in rice at the seedling stage than in other plants across different developmental stages in response to LN stress. In support of this model, heterologous overexpression of *AtGS1* and *AtGS2* in tobacco was shown to increase low-N tolerance by improving growth and nitrogen assimilation [40], providing direct evidence that *GS2* activity can increase NUtE. Our data lead to a testable hypothesis that *GS2* (Seita.3G024100) may contribute to NUtE, a premise that requires future functional validation through detailed biochemical or physiological assays.

In addition, several N metabolism and N assimilation enzyme-encoding genes (3 *AMT*s and 2 *GLUs*) were down-regulated in LN-tolerant foxtail millet at the LN level compared with those at the CK level. Moreover, compared with that in LN-sensitive foxtail millet, the expression of 3 *AMT* genes was lower in the LN-tolerant genotypes under the LN level (Fig. 3D), possibly because the external NO_3_^−^ concentration decreased. Therefore, to maintain nitrogen homeostasis, plants must precisely coordinate nitrate uptake with the activity of core metabolic enzymes. This fine-tuning is exemplified by the high-affinity ammonium transporters *AMT1.1*, *AMT1.2*, and *AMT1.3* in rice, which are rapidly down-regulated under N deficiency to restrict ammonium influx and likely facilitate metabolic rebalancing [41]. *OsAMT1.1* was constitutively expressed in the shoots and roots at both low and high N levels. *OsAMT1.2* and *OsAMT1.3* are nitrogen-inducible and have root-specific expression patterns [42]. *GLU* is involved in the primary assimilation of ammonia in shoots, especially at high NO_3_^−^ levels [43]. Genome-wide association analysis revealed a superior haplotype of *OsGLU1.1*, which presented increased GLU activity and NUtE [44]. Coexpression of *OsGLU1.2* and *OsAMT1.2* could increase NUtE and increase rice yield under nitrogen limitation [45]. Therefore, the differential expression of nitrogen metabolism genes may contribute to the observed disparity in NUtE between foxtail millet genotypes. These coordinated expression changes lead us to hypothesize that they are functionally connected to *SiLNR1* in associating with physiological improvements.

### The nitrogen regulatory protein P-II is involved in contributing to NUtE

The dissection of complex traits can be effectively approached through a layered experimental strategy. This study employed a sequential filtering approach, moving from genetic linkage to transcriptional evidence, to prioritize candidate genes. Meng et al. [46] identified the molecular regulators of NUtE in wheat through an integrated transcriptomic, proteomic, and physiological profiling approach under both high and low N levels. Previously, these approaches have successfully elucidated key aspects of nitrogen regulation. For example, integrated genomic and transcriptomic analyses in rice revealed that drought stress suppresses nitrogen assimilation via the *DST*-*NRT1.2* module [47], whereas similar strategies have identified NUtE-associated genes in rapeseed [48] and revealed a decrease in fruit quality under high nitrogen in apple [49]. Collectively, these studies underscore the efficacy of a stepwise genetic and functional genomics approach for deciphering the complex regulation of nitrogen responses, providing a validated methodological foundation for our study in foxtail millet. The sequential filtering approach enables fine mapping of candidate genes, which could reduce functional validation workflows and increase the feasibility of identifying key candidate genes with both allelic variations and differential expression patterns. This approach provides comprehensive insights into the genotype-specific molecular mechanisms underlying NUtE differences in foxtail millet. This study identified 166 genes with SNP nonsynonymous mutations via BSA-seq. Under low N levels, 14,664 DEGs were identified via RNA-seq in the leaves of both LN-tolerant and LN-sensitive foxtail millet. Six candidate genes were ultimately obtained via combined BSA-seq and RNA-seq analyses.

Among the 6 candidate genes, the expression level of nitrogen regulatory protein P-II (*SiLNR1*, Seita.3G051900) in LN-tolerant foxtail millet under LN conditions was approximately 30 times greater than that in LN-sensitive foxtail millet (Table S3), indicating that Seita.3G051900 (*SiLNR1*) contributes to N metabolism and N uptake. Importantly, the *SiLNR1* allele from Yugu28 not only confers physiological tolerance but also translates into a substantial and measurable yield gain under LN field conditions, solidifying its value as a target for molecular breeding (Table 2).

Similar expression levels of N regulatory protein P-II (*GLNB1*) have been reported in both *Arabidopsis* and tea plants, with higher expression in the N-efficient genotype than in the N-inefficient genotype under N deficiency stress. It is considered a key candidate gene for N metabolism [50,51]. The P-II protein family, initially identified in bacteria and archaea as key regulators of nitrogen metabolism, has homologs in plants [52]. In *Lotus japonicus*, the overexpression of the *GLNB1* gene, which encodes the P-II protein, affects nodule activity under permissive LN conditions and increases nodule numbers at high nitrogen levels, suggesting that P-II is involved in signaling the nutritional status of nitrogen and affecting legume predisposition for nodule formation [53]. Converging evidence from multiple species underscores that P-II (*GLNB1*) may be associating with nitrogen homeostasis. In *Arabidopsis*, P-II coordinates the carbon/nitrogen status and enhances high-affinity N uptake [50], whereas its overexpression elevates the antioxidant capacity via *APX1*-mediated ascorbate accumulation under N stress [51]. Conversely, P-II depletion in *Medicago* disrupts nodulation and N metabolism, severely compromising NUtE [54]. Despite these advances, its mechanism in cereal crops, particularly under LN conditions, remains a critical knowledge gap. Therefore, the identified nitrate-regulating protein P-II-related gene Seita.3G051900 (a LN regulatory gene) will be further studied as a vital gene contributing to NUtE.

### *SiLNR1* coordinates nitrogen acquisition and utilization

Numerous reports have demonstrated that the nitrogen regulatory protein P-II in bacteria plays a primary role in nitrogen metabolism. Several studies have shown that P-II enzymes in plants are involved in regulating the absorption of nitrogen [55]. In this study, 1 of the 6 candidate genes was nitrogen regulatory protein P-II (*SiLNR1*, Seita.3G051900), which corresponds to the rice homologous gene Os05g04220.1 (*GlnB*) and is annotated as a *PII_GLNB* domain that regulates nitrogen utilization. The transcriptome results revealed that *SiLNR1* was highly expressed in the leaves of foxtail millet (Fig. 4). Because *SiLNR1* is highly expressed in the leaves, especially at the fifth leaf stage (Fig. 7B), it was speculated that *SiLNR1* might be expressed in the leaves. The leaf is the organ with the greatest degree of N allocation, and most N in the leaf is directly involved in photosynthetic activities. The NUtE of leaves may influence whole-plant NUtE and, consequently, plant growth [56], which suggests that *SiLNR1* may modulate N utilization and metabolism. Our genetic evidence demonstrated that *SiLNR1* contributes to NUtE and plant growth positively. The results of the phenotype analysis of the *SiLNR1*-overexpressing plants revealed that the primary root length and plant height under both LN and CK conditions were significantly greater than those of the WT plants (Fig. 6 and Fig. S6), which is consistent with the phenotype of nitrogen regulatory protein P-II-related gene overexpression reported in recent studies under low-N stress [53]. Functional analyses demonstrated that *SiLNR1* overexpression significantly increased both nitrogen accumulation and NUtE in foxtail millet (Fig. 10). Consistent with this gain-of-function phenotype, loss-of-function *silnr1* mutants presented markedly inhibited root growth, plant height, and N accumulation under both LN and CK conditions (Fig. 8 and Fig. S7), providing complementary genetic evidence that *SiLNR1* contributes to nitrogen economy positively. Importantly, our functional validation was performed in the Ci846 genetic background, which differs from the discovery germplasms. Future efforts to introgress the identified alleles into elite varieties will be crucial to confirm their agronomic value in different genetic contexts.

Previous studies have shown that genes encoding P-II proteins are up-regulated by low carbon concentrations and are involved in the regulation of N utilization under low-N stress in plants [57,58]. On the basis of our genetic and expression data, we propose that *SiLNR1*, as a nitrogen regulatory P-II protein, may be associated with a previously unreported role in nitrogen metabolism beyond its known function in carbon fixation. This novel function is evolutionarily conserved, as evidenced by the similar role of *CsGLB1* in tea plants [59]. Meanwhile, the expression of *SiNRT1.1* and *SiGS2* associating with nitrogen transporters and assimilatory enzymes showed significant difference among WT plants, *SiLNR1-OE* plants, and *silnr1* mutants under control conditions (Fig. 9C and D). Future work employing chromatin immunoprecipitation followed by sequencing or quantitative PCR with an anti-*SiLNR1* antibody will be crucial for determining whether *SiLNR1* binds directly to these nitrogen metabolism genes and thus acts as a direct transcriptional regulator. The expression of these genes in the *silnr1* mutant background under LN stress is essential for definitively establishing the role of *SiLNR1* responding to nutrient limitation.

We conducted a comparative analysis of the corresponding amino acid sequences of *SiLNR1* from LN-tolerant and LN-sensitive foxtail millet genotypes. The findings revealed 4 amino acid deletions within the coding DNA sequence (coding DNA sequence) region of the *SiLNR1* gene in these 2 genotypes. Notably, none of these mutation sites were located outside the conserved domain, as illustrated in Fig. S4A and B. Canonical PII proteins are known to be regulated by uridylylation [52]. In this study, through the prediction of amino acid phosphorylation sites, we found that 2 specific mutations resulted in marked alterations in protein function. In Yugu28, there was an increase in Ser67, and in Qiyehuang, alanine (Ala59) was transformed into Ser59. These 2 residues, Ser59 in Yugu28, are recognized as common phosphorylation sites (Fig. S4C). Our data, combined with bioinformatic predictions, led us to propose a working model in which *SiLNR1* function might be regulated posttranslationally, potentially through phosphorylation at predicted sites such as Ser59/Ser67. Future studies on phosphorylation-dependent regulatory mutagenesis are needed to confirm the causal role of these phosphorylation sites. This observation provides a compelling explanation for our earlier findings, which demonstrated that LN-tolerant foxtail millet genotypes possess greater N absorption capacity than their LN-sensitive counterparts do [31]. The potential involvement of TOR kinase, as indicated by preliminary Yeast Two-Hybrid (Y_2_H) data, offers a promising avenue for future research to identify the upstream regulators of *SiLNR1*. To validate this hypothesis, direct protein–protein interactions remain to be experimentally validated to identify protein kinases capable of phosphorylating *SiLNR1*. Our findings suggest that *SiLNR1* is associated with enhanced NUtE and plant growth in foxtail millet. Therefore, targeted gene editing of *SiLNR1* could be explored as a strategy for developing foxtail millet germplasm with improved NUtE. Finally, a complete understanding of the *SiLNR1*-associated regulatory pathway remains to be established, and future genome-wide studies are needed to identify its upstream regulators and downstream target genes.

### *SiLNR1* as a supported candidate: Limitations and future directions

The identification of *SiLNR1* associated with LN tolerance provides valuable genetic resources and potential candidate targets for future molecular breeding efforts, including the development of gene-edited lines with improved NUtE. While our integrated approach provides strong evidence for the role of *SiLNR1*, we acknowledge 2 limitations that define the scope of our current conclusions. First, fine-mapping within the RIL population to pinpoint the exact causal polymorphism was not performed. Second, a formal genetic complementation assay of the *silnr1* mutant with the WT allele remains to be conducted. Nevertheless, the candidacy of *SiLNR1* is underscored by the convergence of multiple independent lines of evidence: its colocalization with a major QTL, perfect correlation of its natural alleles with agronomic traits of the RIL population, opposing and severe phenotypes in both gain-of-function and loss-of-function transgenic lines in the same background, and its association with the expression of core nitrogen metabolism genes. Therefore, we present *SiLNR1* as a strongly supported, high-priority candidate gene.

While our study suggests *SiLNR1* in LN tolerance, several key aspects of its molecular function remain to be elucidated. First, direct evidence for a physical interaction between *SiLNR1* and TOR kinase, as well as the functional significance of the predicted phosphorylation sites, requires validation through coimmunoprecipitation, Y_2_H, and phospho-mutagenesis assays. Second, it is currently unknown whether *SiLNR1* directly binds to the promoters of downstream genes such as *NRT2.1* and *AMT1.1* to regulate their transcription or whether this regulation is indirect. These important questions represent the primary focus of our ongoing and future research.

## Materials and Methods

### Plant materials

The LN-tolerant foxtail millet genotype “Yugu28” was selected as the female parent, whereas the LN-sensitive genotype “Qiyehuang” was selected as the male parent. A total of 120 lines from their RIL populations were developed via single-seed descent to the F_8_ generation in this study.

### Field experiment

A RIL population comprising 120 lines derived from reciprocal crosses between the LN-tolerant female parent Yugu28 and the LN-sensitive male parent Qiyehuang was cultivated under field conditions. Field trials were conducted at the Modern Agriculture Research and Development Base in Xinxiang, Henan Province (34.55°N, 113.36°E, China), from June to September of 2022, 2023, and 2025. The experiment was established within a long-term nitrogen fertilization field initiated in 2012. The experimental soil exhibited the following physicochemical properties: organic matter, 2.35 g·kg^−1^; total nitrogen, 0.71 g·kg^−1^; Olsen-P, 16.25 mg·kg^−1^; and total potassium, 37.41 mg·kg^−1^. Two nitrogen treatments, LN (no nitrogen) and T_1_ (175 kg·N·ha^−1^ applied as urea at sowing), were applied as controls. The experiment was arranged in a randomized complete block design with 3 biological replicates. Each plot for a RIL measured 4 m^2^ (0.8 m × 5.0 m) with a planting density of 600,000 plants per hectare. All field management practices, including pest and weed control, followed standard high-yield practices. At maturity, 15 individual plants were randomly sampled from each plot for subsequent phenotypic, NUtE, and yield trait measurements.

Plants were partitioned into shoots (stem/leaf), roots, and seeds. All the tissues were oven-dried at 70 °C to a constant weight for dry weight measurement. The total nitrogen content was determined for each tissue via the Dumas combustion method with an elemental analyzer. The whole-plant nitrogen content was calculated as the sum of the nitrogen contents from all the tissues. NUtE was calculated as the ratio of total plant dry weight to whole-plant nitrogen content, according to Moll et al. [60,61].

### Hydroponic experiments

Foxtail millet genotypes, including the LN-tolerant Yugu28, the LN-sensitive Qiyehuang, and their RIL populations, were hydroponically cultivated under controlled conditions. A 16/8-h light/dark photoperiod with a light intensity of 150-μmol photons·m^−2^·s^−1^ provided by white light-emitting diode lamps, a constant temperature of 28/26 °C day/night, and a relative humidity of 70% ± 5% was used. At 7 days postgermination (DPG), the plants were subjected to modified Hoagland nutrient solutions [KH_2_PO_4_, 0.2 mmol·l^−1^; MgSO_4_, 1.0 mmol·l^−1^; KCl, 1.5 mmol·l^−1^; CaCl_2_, 1.5 mmol·l^−1^; FeEDTA, 0.1 mmol·l^−1^; H_3_BO_3_, 1 × 10^−3^ mmol·l^−1^; (NH4)_6_Mo_7_O_24_, 5 × 10^−5^ mmol·l^−1^; CuSO_4_, 5 × 10^−4^ mmol·l^−1^; ZnSO_4_, 1 × 10^−3^ mmol·l^−1^; and MnSO_4_, 1 × 10^−3^ mmol·l^−1^] with contrasting nitrate regimes: LN treatment [LN; Ca(NO3)_2_, 0.25 mmol·l^−1^; NO_3_^−^, 0.5 mmol·l^−1^] and the control [CK; Ca(NO_3_)_2_, 1.25 mmol·l^−1^ and NO_3_^−^, 2.5 mmol·l^−1^]. Nutrient solutions were replenished every 2 d to maintain ionic equilibrium. Following a 21-d treatment period, root and shoot tissues were harvested for transcriptome profiling via RNA-seq to dissect genotype-specific nitrogen response networks.

### Evaluation of NUtE and BSA-seq analysis

After maturity, the NUtE of parents and 120 lines of the RIL population were measured. Two parents and each line of the RIL population selected 15 plants to measure their NUtE. SPSS software was used to analyze the NUtE of 120 groups, and Excel software was used to select 15 strains of low NUtE and 15 strains of high NUtE to construct an extreme mixing pool.

For BSA-seq, young leaves from parental lines (Yugu28 and Qiyehuang) and RILs were flash-frozen in liquid nitrogen and stored at −80 °C. Genomic DNA was extracted via a modified cetyltrimethylammonium bromide protocol [62],with equal-molar pooling of 15 extreme high-NUtE (RILH pool) and 15 low-NUtE (RILL pool) lines. Whole-genome resequencing (150 bp paired-end) was performed by JiZhi Biotech (Tianjin, China) on the Illumina NovaSeq 6000 platform, which was aligned to the *Setaria italica* v2.2 reference genome (https://phytozome-next.jgi.doe.gov/info/Sitalica_v2_2).

Variant detection (SNPs, and Insertions and Deletions [InDels]) was performed via the Sentieon Genomics Suite [63] following a Genome Analysis Toolkit best-practices-compliant workflow. The preprocessing steps included duplicate marking (Picard MarkDuplicates) to remove PCR artifacts, local realignment (Sentieon TNscope) around InDel regions to minimize mapping errors, and base quality score recalibration to correct systematic sequencing biases. High-confidence variants were identified through the joint calling of SNPs and InDels via the Sentieon Haplotyper, with stringent filtering parameters (QUAL ≥ 30, DP ≥ 10, GQ ≥ 20) applied to ensure allele call accuracy. The final variant annotation was performed against the reference genome via SnpEff v5.1, and only homozygous and heterozygous calls with Mendelian consistency across pools were retained.

The SNP frequencies (SNP index) of the 2 extreme mixing pools were calculated following the method of Fekih et al. [64], using parents as references. In order to reduce the impact of sequencing errors and alignment errors, we filtered the polymorphic loci of parents after calculating the SNP index and filtered out the loci with a SNP index that was less than 0.3 and a SNP index depth less than 7 in both pools, as well as the loci with an index missing in any pool. The ΔSNP index was calculated as (SNP-index_High − SNP-index_Low). A genome-wide significance threshold was determined using a permutation test (*n* = 1,000 iterations, *P* < 0.01). For each permutation, the genome-wide maximum |ΔSNP index| was recorded to generate a null distribution. The 95th percentile of this distribution (*P* < 0.05) was used as the genome-wide significance threshold. Regions where the ΔSNP index consistently exceeded this threshold were considered candidate regions.

### Transcriptome and qRT-PCR analysis

Transcriptomic profiling was performed on LN-tolerant Yugu28 and LN-sensitive Qiyehuang genotypes under control (CK: 2.5 mmol·l^−1^ NO_3_^−^) and LN (0.5 mmol·l^−1^ NO_3_^−^) conditions, with 3 biological replicates per genotype × treatment combination (*n* = 6). Total RNA was isolated via the RNeasy Plant Mini Kit (Qiagen, catalog no. 74904), and its integrity was verified via an Agilent 2100 Bioanalyzer (RNA integrity number ≥ 8.0). Total RNA was quantified via a Qubit 2.0 fluorometer (Thermo Fisher). Subsequently, strand-specific RNA-seq libraries were constructed with the NEBNext Ultra II RNA Library Prep Kit (NEB, E7770S). Finally, the prepared libraries were sequenced on an Illumina NovaSeq 6000 platform to generate 150-bp paired-end reads, with a target depth of 40 million reads per sample.

Transcript abundance was estimated via Salmon (v1.9.0) with default parameters and normalized to the fragments per kilobase of transcript per million mapped reads value. Differential expression analysis was subsequently performed with DESeq2, identifying genes with an absolute log2-fold change greater than 1 and an FDR-adjusted *P* value less than 0.05. Finally, the functional profiles of these DEGs were investigated through GO and KEGG pathway enrichment analyses via the clusterProfiler package (v4.0).

For qRT-PCR validation, 9 DEGs spanning nitrogen metabolism pathways were selected. The primers (Table S1) were designed via Primer-BLAST (National Center for Biotechnology Information [NCBI]) with a *T*m of 60 ± 2 °C and amplicon lengths of 80 to 150 bp. qRT-PCR assays were conducted in triplicate on a Bio-Rad CFX96 Touch system using SYBR Premix Ex Taq II (Tiangen, FP209). The thermal cycling protocol consisted of initial denaturation at 95 °C for 30 s, followed by 40 cycles of 95 °C for 5 s and 60 °C for 30 s. Primer specificity for each reaction was verified by melting curve analysis from 65 to 95 °C with 0.5 °C increments. The relative gene expression levels were calculated via the 2^−ΔΔCt^ method and normalized to the *Siactin* gene (Seita.1G215400), which demonstrated stable expression across all experimental conditions (coefficient of variation < 5%) [65].

### Transcriptome analysis under different conditions

The raw and processed RNA-seq data generated in this study have been deposited in the NCBI Sequence Read Archive under BioProject accession PRJNA1083237 (https://www.ncbi.nlm.nih.gov/bioproject/PRJNA1083237), adhering to FAIR (Findable, Accessible, Interoperable, Reusable) data principles. These condition-specific transcriptomic profiles enable the systematic exploration of genotype-by-nitrogen treatment interactions through high-throughput gene expression dynamics.

### Identification of candidate genes

Candidate genes governing NUtE were systematically identified through an integrative multiomics pipeline: Nonsynonymous SNPs/InDels linked to NUtE divergence were mapped via BSA-seq via a Euclidean distance algorithm (≥0.95), and the SNP index threshold (ΔSNP ≥ 0.60, *P* < 0.01) had moderate-or high-impact SNP effect sizes. Differential expression analysis (|log2FC| > 3, FDR < 0.01) of prioritized loci was performed across LN-tolerant (Yugu28) and LN-sensitive (Qiyehuang) genotypes under nitrogen stress, and final candidates were selected on the basis of tissue-specific expression patterns, functional annotation, and evolutionary conservation.

### Subcellular localization

To resolve subcellular localization dynamics, the coding sequence of the candidate gene was PCR-amplified (primers in Table S1) and cloned and inserted into the pAN580 plant expression vector via Gateway LR recombination, generating an N-terminal green fluorescent protein (GFP) fusion under the CaMV 35S promoter. Rice protoplasts were isolated from 14-d-old seedlings and transfected with the GFP fusion construct alongside a nuclear marker (red fluorescent protein [RFP]-tagged histone 2B) via polyethylene glycol-mediated transformation as described previously [66]. After 16 to 18 h of incubation in darkness (25 °C), fluorescence signals were captured via a Nikon C2-ER laser scanning confocal microscope equipped with GaAsP detectors. GFP was excited at 488 nm (emission: 505 to 530 nm), and RFP was excited at 561 nm (emission: 570 to 620 nm). Z-stack images were processed via NIS-Elements AR 5.21 software (Nikon) to confirm signal colocalization, which was quantified via Pearson’s correlation coefficient (>0.8 for nuclear specificity).

### Genetic transformation and phenotypic identification of foxtail millet

To investigate gene function, we generated both overexpression and knockout lines in the foxtail millet cultivar Ci846. For overexpression, the candidate gene was cloned and inserted into the pBWA(V)HS vector, which was subsequently introduced into foxtail millet via *Agrobacterium tumefaciens*-mediated transformation of secondary embryogenic calli [67]. Putative transgenic plants were initially selected on the basis of antibiotic resistance. Finally, *SiLNR1*-overexpressing transgenic plants were confirmed via qRT-PCR, and homozygous individuals were advanced for subsequent functional analyses.

For knockout mutagenesis, pYLCRISPR was used to construct a CRISPR vector targeting *SiLNR1*, which was subsequently employed for genetic transformation of the foxtail millet variety Ci846. CRISPR-P 2.0 was utilized to design the base-pairing sequence of the single guide RNA (sgRNA) (5′-ATGTCGCCAGCGACCTCCGC-3′) targeting a single exon of *SiLNR1*. The sgRNA expression cassettes were driven by the promoter. The construct was assembled via Golden Gate assembly and verified via Sanger sequencing before transformation. To complement the *silnr1* mutant, genomic fragments of *SiLNR1* (884 bp) were cloned and inserted into the *pRGEB32* vector. The resulting construct was subsequently transformed into *A. tumefaciens* EHA105, which was subsequently transformed into a foxtail millet callus (Ci846) induced from mature seeds of the *silnr1* mutant. Genomic DNA was extracted from T_0_ transgenic plants and their T_1_/T_2_ progeny. The target region was amplified with gene-specific primers (forward: GTCGCCAGCGACCTCCGCCACT; reverse: CGCGCTCTGGGCGCGCACGG). The PCR products were sequenced, and the resulting chromatograms were analyzed via DECODR to identify insertion/deletion mutations. Homozygous T_2_ lines, which presented a single, clear indel mutation in the sequencing chromatogram and segregated no WT allele in the T_1_ generation, were propagated for subsequent phenotypic evaluation. Potential off-target sites were predicted in the *Setaria italica* reference genome (v2.2) via Cas-OFFinder. No off-target mutations were detected at the analyzed sites.

We sterilized the surface of the seeds via the use of 5% (v/v) sodium hypochlorite, after which they were thoroughly rinsed with sterile water. To synchronize germination, we then stratified the seeds at 26 °C for 48 h in complete darkness. Uniformly germinated seeds were transferred to our experimental media: half-strength nitrogen-free Murashige and Skoog medium (Sangon Biotech, Shanghai) supplemented with 1% sucrose solidified with 0.8% agar (Solarbio, Beijing), with a final pH of 5.8. The pots were placed in a controlled-environment growth chamber (Conviron) at 28 °C with a 16-h photoperiod (26 °C, 8-h dark), a photosynthetic photon flux density of 150 μmol·m^−2^·s^−1^, and 70% ± 5% of relative humidity. At 10 DPG, primary root length, shoot height, and tissue-specific nitrogen content (shoot versus root) were quantified across 2 nitrate regimes: LN (0.5 mmol·l^−1^ NO_3_^−^) and the control (CK, 2.5 mmol·l^−1^ NO_3_^−^).

Foxtail millet plants were cultivated in a hydroponic-vermiculite hybrid system to simulate field-like nitrogen responses. Following sterilization, the seeds were aseptically transferred onto sterile moist filter paper for germination (25 °C, 16-h photoperiod) and transplanted into 30 cm × 25 cm pots filled with prewashed horticultural-grade vermiculite (particle size, 2 to 4 mm) and field soil, and the weight ratio was 1:3. We arranged the pots in trays filled with modified Hoagland solution, employing a subirrigation system to ensure consistent nutrient availability to the roots [basal composition: 0.2 mmol·l^−1^ KH_2_PO_4_, 1.0 mmol·l^−1^ MgSO_4_, 1.5 mmol·l^−1^ KCl, 1.25 mmol·l^−1^ Ca(NO_3_)_2_, 1.5 mmol·l^−1^ CaCl_2_, 0.1 mmol·l^−1^ FeEDTA, and micronutrients], adjusted to a pH of 5.8. At 21 d posttransplantation (vegetative stage V3), the plants were divided into 2 nitrogen levels: control (CK, continuous 2.5 mmol·l^−1^ NO_3_^−^) and LN (0.5 mmol·l^−1^ NO_3_^−^). Nutrient solutions were replenished every 72 h to maintain ionic stability. At the maturity stage, the plants were partitioned into seed, shoot, and root tissues. Root length and plant height were measured from 3 biological replicates per genotype/treatment and analyzed via SPSS software v26.0. The NUtE was calculated according to the “Field experiment” section.

### Experimental replication and statistical analysis

All experiments included 3 biological replicates, with the data presented as the means ± standard error of the mean (SEM). The assumptions of normality (Shapiro–Wilk test, *P* > 0.10) and homogeneity of variances (Levene’s test, *P* > 0.05) were confirmed prior to parametric analyses. For multigroup comparisons, 1-way analysis of variance (ANOVA) was conducted via SPSS Statistics (v26.0, IBM), with post hoc Tukey’s honestly significant difference test applied to determine specific group differences. Pairwise comparisons between 2 conditions (e.g., LN versus CK) were performed via a 2-tailed unpaired Student *t* test. Statistical significance was defined as *P* < 0.05. To provide a complete interpretation of the results, effect sizes (Cohen’s *d* for *t* test; partial η^2^ for ANOVA) alongside their 95% confidence intervals are detailed in Table S2.

## Ethical Approval

This article does not contain any studies with human participants or animals performed by any of the authors. All transgenic work was conducted in greenhouse facilities in accordance with our institutional biosafety protocols.

## Data Availability

The transcriptome data used in the present study are available in the NCBI Sequence Read Archive database (https://www.ncbi.nlm.nih.gov/sra) (accession number: PRJNA1083237). All datasets are publicly available for noncommercial research use via the GEO official portal. The other data that support the findings of this study are available on request from the corresponding author.
