# Supplementary material for: SiLNR1-Mediated Nitrogen Regulatory Signaling Enhances Nitrogen Use Efficiency and Grain Yield in Foxtail Millet (Setaria italica L.) under Low-Nitrogen Stress
Source: Research (Wash D C). 2026 Feb 25;9:1148. doi: 10.34133/research.1148 (PMC12932939; doi:10.34133/research.1148)
Supplement: Supplementary 1 — Figs. S1 to S7 Tables S1 to S6 [file research.1148.f1.zip › supplement figures and tables-12.31.docx]

***cSiLNR1*-Mediated Nitrogen Regulatory Signaling Enhances Nitrogen Utilization Efficiency (NUtE) and Grain Yield in Foxtail Millet (*Setaria italica* L.) Under Low Nitrogen Stress**

Supplementary materials

Table S1. Primer sequence used in this study.

| **Gene name** | **Forward primer** | **Reverse primer** | **Description** |
| --- | --- | --- | --- |
| *SiLNR1* | ATGTCGCCAGCGACCT | TAAGATTGACACAACAGAGGACAG | Gene Cloning |
| *SiLNR1* | GGGGATTAGAGGTGTGACGG | TACTGCTTCGACCTGCTCCT | qRT-PCR |
| *SiACTIN* | GGATACTCTTTCACCACCTC | ACCTCAGGGCACCTAAAC | qRT-PCR |
| Seita.1G300600 | TGCTACATTTCCCACGACCC | CTAGTGGCGGAGGTGAGAGT | qRT-PCR |
| Seita.3G024100 | AGCTGGAGTTGTGCTTACCC | ATGGCGCAGAGAAAGGTTCA | qRT-PCR |
| Seita.3G051900 | CGTCCTCATCCGCCAATCTC | CTGAGAGAACGACTGGGTGG | qRT-PCR |
| Seita.5G052700 | ATCAGACCCAAAGGGGGAGA | CAGCATACAATCAGGGGCCA | qRT-PCR |
| Seita.6G020900 | CTCCTACTTCGGCTTCTGCG | ATTGGCAGGATTGGAGGAGC | qRT-PCR |
| Seita.9G477200 | GTTACATCTTAGTGACTCAATGCTT | GAACCACCCTTCAGGAACGA | qRT-PCR |
| Seita.1G186500 | GGAGTATCTGGCGTCAAGGG | CGTAGCCCTTCCTGTTGAGG | qRT-PCR |
| Seita.4G124800 | TTCTCTGTCGTGACCGAACCTT | GAACCGAAGGCCGAGAGAG | qRT-PCR |
| Seita.8G065700 | GCTTCCGGGTGGAGATGTCA | GTGTAGTAGATGACGAAGGCGA | qRT-PCR |
| *SiAAT1.1* | AGAGGAGCCATCTGTTCTGC | AGCACCAAGGAGAAGGGGTA | qRT-PCR |
| *Sizf-DOF1* | TGCTGGGCTCTAGAGTGGAT | TTCGACTGGTTGGCTGTACC | qRT-PCR |
| *SiGS2* | AACCTTTCTCTGCGCCATGA | GTTTCTGCCAGTAGCCCTGT | qRT-PCR |
| *SiNRT1.1* | GTGTCGGAGCTCTTCACCAA | TGTCCTTGTCGAGGTCGTTG | qRT-PCR |
| *SiNRT1.3* | CTGGCATCCAGGGAGCAAAA | AGCATTGAGTCACTAAGATGTAACT | qRT-PCR |
| *Sizf-DOF2* | CACTTCCTGCAGAGGGTTCG | TATCCGGATTGTCTTCGGCG | qRT-PCR |
| *SiAAT2.1* | CTGGGGAACATTGCCTTTGC | GAGCCTATACCCACTGCCAC | qRT-PCR |
| *SiAAT2.3* | GGGTGGAGATGTCAACGAAGG | GGTCGAAGTCCGGTATCTGC | qRT-PCR |
| *SiGST1* | CGGGCTTTGTTCTGCCATTT | CGATGTGGTGACAGTTGGGT | qRT-PCR |

Table S2. The quality summary of BSA-Seq data.

| **Sample** | **Raw base**  **(bp)** | **Clean Base**  **(bp)** | **Effective Rate**  **(%)** | **Error Rate**  **(%)** | **Q30**  **(%)** | **Q20**  **(%)** | **GC**  **(%)** |
| --- | --- | --- | --- | --- | --- | --- | --- |
| P1 | 11,548,383,000 | 10,985,236,216 | 95.12 | 0.02 | 90.85 | 96.4 | 49.57 |
| P2 | 11,851,859,700 | 11,151,562,452 | 94.09 | 0.02 | 90.63 | 96.07 | 49.81 |
| RIL (L) | 12,658,258,800 | 12,132,868,942 | 95.85 | 0.01 | 92.67 | 96.98 | 48.75 |
| RIL (H) | 10,914,364,200 | 10,533,621,434 | 96.51 | 0.01 | 93.65 | 97.47 | 46.61 |

Table S3**.** Quality statistics of mapping with the reference genome for BSA-Seq.

| **Sample** | **Mapped Reads** | **Total**  **Reads** | **Mapping Rate(%)** | **Average depth（X）** | **Coverage 1X(%)** | **Coverage 4X(%)** |
| --- | --- | --- | --- | --- | --- | --- |
| Yugu28 | 72,847,379 | 73,801,078 | 98.71 | 23.11 | 96.15 | 93.94 |
| Qiyehuang | 74,130,966 | 74,966,426 | 98.89 | 23.49 | 96.15 | 93.77 |
| RIL (H) | 81,499,597 | 82,136,912 | 99.22 | 24.82 | 97.58 | 96.26 |
| RIL (L) | 70,536,432 | 71,188,002 | 99.08 | 22.46 | 96.95 | 95.94 |

**Total Reads:** Final sequencing output. **Mapped Reads:** Reads uniquely aligned to the reference. **Mapping Rate:** Percentage of aligned reads. **Average Depth:** Mean base coverage (total aligned bases / genome size). **Coverage ≥1×:** Fraction of genome covered at ≥1× depth. **Coverage ≥4×:** Fraction of genome covered at ≥4×depth.

Table S4. The results of SNP annotation.

| **Total** | **Intergenic** | **Upstream** | **Downstream** | **UTR3** | **UTR5** | **Intronic** | **Splicing** | **Exonic** | | | |
| --- | --- | --- | --- | --- | --- | --- | --- | --- | --- | --- | --- |
|  |  |  |  |  |  |  |  | **Stop gain** | **Stop loss** | **Synonymous** | **Non-**  **synonymous** |
| 2,072,744 | 1,554,143 | 117,808 | 99,026 | 31,059 | 17,492 | 13,6113 | 486 | 1,145 | 165 | 37,079 | 57,798 |

Table S5. The basic situation of 166 candidate genes.

| **Serial**  **Number** | **Ensembl Gene Id** | **Chromosome** | **Snp Eff** | **Δ(SNP-index)** | **Start position** | **End position** | **Description** |
| --- | --- | --- | --- | --- | --- | --- | --- |
| 1 | Seita.2G324200 | II | L | -0.69 | 41215989 | 41216982 | UDP-glucoronosyl and UDP-glucosyl transferase domain containing protein |
| 2 | Seita.2G324400 | II | L | -0.82 | 41225357 | 41226201 | anthocyanidin 5,3-O-glucosyltransferase |
| 3 | Seita.2G325000 | II | L | -0.74 | 41278317 | 41278641 | SPL13 - SBP-box gene family member |
| 4 | Seita.2G325200 | II | L | -0.71 | 41292914 | 41293813 | expressed protein |
| 5 | Seita.2G325500 | II | L | -0.68 | 41314811 | 41315654 | retrotransposon protein |
| 6 | Seita.2G325600 | II | L | -0.71 | 41317209 | 41320895 | WD domain, G-beta repeat domain containing protein |
| 7 | Seita.2G325800 | II | L | -0.82 | 41330624 | 41332140 | targeting protein-related |
| 8 | Seita.2G326500 | II | L | -0.63 | 41398984 | 41400880 | mitotic checkpoint serine/threonine-protein kinase BUB1 |
| 9 | Seita.2G327200 | II | L | -0.66 | 41475895 | 41476561 | dimethyladenosine transferase |
| 10 | Seita.2G327900 | II | L | -0.81 | 41520813 | 41521244 | endonuclease/exonuclease/phosphatase family domain containing protein |
| 11 | Seita.2G328000 | II | L | -0.77 | 41522924 | 41531233 | expressed protein |
| 12 | Seita.2G328100 | II | L | -0.73 | 41536284 | 41539042 | monocopper oxidase |
| 13 | Seita.2G328900 | II | L | -0.73 | 41604399 | 41604854 | terpene synthase |
| 14 | Seita.2G329300 | II | L | -0.89 | 41626950 | 41629816 | chorismate mutase/prephenate dehydratase |
| 15 | Seita.2G332000 | II | L | -1.0 | 41802540 | 41802873 | MATE efflux family protein, putative |
| 16 | Seita.2G332400 | II | L | -0.76 | 41824939 | 41825104 | RNA recognition motif containing protein |
| 17 | Seita.2G332700 | II | L | -0.82 | 41833189 | 41833330 | seed maturation protein PM23 |
| 18 | Seita.2G333000 | II | L | -0.67 | 41849417 | 41856087 | IQ calmodulin-binding motif family protein |
| 19 | Seita.2G333400 | II | L | -0.76 | 41881983 | 41882447 | AAA family ATPase |
| 20 | Seita.2G334000 | II | L | -0.75 | 41918684 | 41919054 | cytochrome P450 |
| 21 | Seita.2G334900 | II | L | -0.67 | 41954843 | 41956027 | DUF617 domain containing protein |
| 22 | Seita.2G335400 | II | L | -0.67 | 41989594 | 42014387 | glutamate receptor |
| 23 | Seita.2G336300 | II | L | -0.78 | 42049152 | 42051128 | auxin-induced protein 5NG4 |
| 24 | Seita.2G336800 | II | L | -0.61 | 42097372 | 42097797 | lipase class 3 family protein |
| 25 | Seita.2G338400 | II | L | -0.70 | 42210458 | 42211268 | expressed protein |
| 26 | Seita.2G339100 | II | L | -0.50 | 42250373 | 42253968 | NUC153 domain containing protein |
| 27 | Seita.2G339400 | II | L | -0.68 | 42263062 | 42264382 | ankyrin repeat family protein |
| 28 | Seita.2G339900 | II | L | -0.82 | 42303391 | 42303620 | aspartic proteinase nepenthesin-1 precursor |
| 29 | Seita.2G340700 | II | L | -0.76 | 42354411 | 42354773 | receptor-like serine-threonine protein kinase |
| 30 | Seita.2G341300 | II | L | -0.64 | 42402407 | 42405530 | TKL_IRAK_DUF26-lc.10 - DUF26 kinases have homology to DUF26 containing loci |
| 31 | Seita.2G341600 | II | L | -0.81 | 42433602 | 42434780 | glucan endo-1,3-beta-glucosidase precursor |
| 32 | Seita.2G342900 | II | L | -0.68 | 42512275 | 42513837 | beta-amylase |
| 33 | Seita.2G344500 | II | L | -0.82 | 42630122 | 42632229 | F-box/RNI-like superfamily protein |
| 34 | Seita.2G348000 | II | L | -0.82 | 42921773 | 42937369 | CSLF3 - cellulose synthase-like family F |
| 35 | Seita.2G358800 | II | L | 0 | 43677641 | 43681047 | xylose isomerase |
| 36 | Seita.2G377800 | II | L | 0 | 44928002 | 45280101 | BRASSINOSTEROID INSENSITIVE 1 precursor |
| 37 | Seita.2G396300 | II | H | 0.79 | 46093620 | 46096612 | Transducin/WD40 repeat-like superfamily protein |
| 38 | Seita.2G397600 | II | H | 0.76 | 46166947 | 46167474 | E3 ubiquitin ligase SCF complex subunit SKP1/ASK1 family protein |
| 39 | Seita.2G398500 | II | H | 0.78 | 46222128 | 46222553 | FAD/NAD(P)-binding oxidoreductase family protein |
| 40 | Seita.2G403400 | II | M | 0.35 | 46511645 | 46513010 | SNARE associated Golgi protein |
| 41 | Seita.2G431100 | II | H | 0.80 | 48183372 | 48183675 | peroxidase precursor |
| 42 | Seita.3G024100 | III | H | 0.67 | 1455306 | 1459782 | Glutamine synthetase |
| 43 | Seita.3G051900 | III | H | 0.85 | 3276811 | 3279806 | nitrogen regulatory protein |
| 44 | Seita.3G080000 | III | H | 0.75 | 5090105 | 5091125 | C-5 cytosine-specific DNA methylase |
| 45 | Seita.3G081300 | III | L | 0.13 | 5198301 | 5200230 | alpha/beta fold family protein |
| 46 | Seita.3G081600 | III | M | 0.41 | 5208093 | 5210612 | TKL_IRAK_DUF26-lc.11 - DUF26 kinases have homology to DUF26 containing loci |
| 47 | Seita.3G081800 | III | M | 0.67 | 5217715 | 5218437 | plant protein of unknown function domain containing protein |
| 48 | Seita.3G082700 | III | M | 0.68 | 5273124 | 5275504 | Auxin efflux carrier family protein |
| 49 | Seita.3G082900 | III | M | 0.52 | 5298305 | 5300031 | cyclin-A1 |
| 50 | Seita.3G083100 | III | M | 0.65 | 5305571 | 5307144 | mitotic checkpoint family protein |
| 51 | Seita.3G083700 | III | M | 0.46 | 5349964 | 5351217 | amino acid transporter |
| 52 | Seita.3G086200 | III | M | 0.52 | 5548821 | 5551248 | lysine histidine transporter 2 |
| 53 | Seita.3G087000 | III | M | 0.50 | 5598202 | 5598447 | 3-oxoacyl-reductase, chloroplast precursor |
| 54 | Seita.3G087400 | III | H | 0.68 | 5622189 | 5623460 | Jacalin-like lectin domain containing protein |
| 55 | Seita.3G087500 | III | H | 0.65 | 5629740 | 5630961 | cytokinin-O-glucosyltransferase 2, putative |
| 56 | Seita.3G087800 | III | M | 0.44 | 5668836 | 5670348 | Zinc finger (C3HC4-type RING finger) family protein |
| 57 | Seita.3G088500 | III | M | 0.51 | 5729507 | 5730610 | expressed protein |
| 58 | Seita.3G088700 | III | M | 0.42 | 5733313 | 5735104 | S-locus lectin protein kinase family protein |
| 59 | Seita.3G089000 | III | M | 0.38 | 5742939 | 5752853 | oxidoreductase, 2OG-Fe oxygenase family protein |
| 60 | Seita.3G089200 | III | M | 0.44 | 5759888 | 5765261 | major facilitator superfamily antiporter |
| 61 | Seita.3G089700 | III | M | 0.47 | 5790751 | 5793682 | aspartyl aminopeptidase |
| 62 | Seita.3G090000 | III | M | 0.53 | 5822662 | 5827005 | zinc finger, C3HC4 type domain containing protein |
| 63 | Seita.3G090300 | III | M | 0.64 | 5843819 | 5866382 | argininosuccinate synthase, chloroplast precursor |
| 64 | Seita.3G091100 | III | L | 0 | 5903329 | 5910850 | histidine triad family protein |
| 65 | Seita.3G386000 | III | H | 0.60 | 48738042 | 48739660 | glutathione S-transferase, C-terminal domain containing protein |
| 66 | Seita.3G388500 | III | L | -0.18 | 48996220 | 48996561 | Plant protein of unknown function |
| 67 | Seita.3G388700 | III | L | 0.25 | 49016642 | 49021242 | NB-ARC domain-containing disease resistance protein |
| 68 | Seita.3G392400 | III | M | 0.63 | 49350161 | 49351383 | MATE efflux family protein |
| 69 | Seita.3G392500 | III | M | 0.67 | 49352912 | 49355067 | PPR repeat containing protein |
| 70 | Seita.3G392800 | III | H | 0.87 | 49366045 | 49367513 | wall associated kinase 4 |
| 71 | Seita.3G393200 | III | M | 0.69 | 49428917 | 49431648 | L-Aspartase-like family protein |
| 72 | Seita.3G394500 | III | L | 0.38 | 49590980 | 49592259 | Protein of unknown function |
| 73 | Seita.3G394800 | III | H | 0.71 | 49617828 | 49618455 | amino acid permease family protein |
| 74 | Seita.3G395100 | III | M | 0.56 | 49647767 | 49648579 | Methyltransferase small domain containing protein |
| 75 | Seita.3G395700 | III | M | 0.68 | 49682911 | 49684822 | NB-ARC domain-containing disease resistance protein |
| 76 | Seita.3G396100 | III | M | 0.69 | 49708802 | 49711576 | NBS-LRR disease resistance protein |
| 77 | Seita.3G396400 | III | H | 0.86 | 49742476 | 49743890 | sodium/calcium exchanger protein |
| 78 | Seita.3G398200 | III | H | 0.73 | 49853387 | 49853644 | Auxin-responsive SAUR gene family member |
| 79 | Seita.3G400300 | III | L | 0.33 | 50020783 | 50024927 | powdery mildew resistance protein PM3A |
| 80 | Seita.3G402100 | III | L | 0 | 50171293 | 50173658 | receptor-like protein kinase HAIKU2 precursor |
| 81 | Seita.3G402900 | III | H | 0.74 | 50240989 | 50241328 | MYB family transcription factor |
| 82 | Seita.3G403900 | III | L | 0 | 50290617 | 50290688 | Glycine and cysteine rich family protein precursor |
| 83 | Seita.3G404300 | III | L | 0 | 50306716 | 50307399 | DNA binding protein |
| 84 | Seita.3G404700 | III | L | 0 | 50323326 | 50324772 | ankyrin repeat domain-containing protein |
| 85 | Seita.4G057300 | IV | H | 1.0 | 4243524 | 4244181 | Calmodulin-related calcium sensor protein |
| 86 | Seita.4G057700 | IV | H | 1.0 | 4282267 | 4287577 | HIRAN domain containing protein |
| 87 | Seita.4G058000 | IV | H | 1.0 | 4303531 | 4307119 | co-factor for nitrate, reductase and xanthine dehydrogenase 5 |
| 88 | Seita.4G058500 | IV | L | 0 | 4327566 | 4329932 | wall associated kinase 5 |
| 89 | Seita.4G268700 | IV | H | 0.83 | 38596357 | 38687750 | glyceraldehyde-3-phosphate dehydrogenase |
| 90 | Seita.4G271200 | IV | M | 0.70 | 38796937 | 38802238 | delta-aminolevulinic acid dehydratase |
| 91 | Seita.4G271400 | IV | H | 0.82 | 38812011 | 38812768 | histone acetyltransferase HAC1 |
| 92 | Seita.4G271700 | IV | M | 0.70 | 38823738 | 38823814 | thylakoid lumenal 16.5 kDa protein |
| 93 | Seita.4G276500 | IV | M | 0.65 | 39294865 | 39294983 | inorganic phosphate transporter |
| 94 | Seita.4G279000 | IV | L | 0 | 39463999 | 3955089 | SAUR-like auxin-responsive protein family |
| 95 | Seita.5G037900 | V | M | 1.0 | 3509608 | 3510823 | plastocyanin-like domain containing protein |
| 96 | Seita.5G049000 | V | M | 1.0 | 4422435 | 4423841 | serpin domain containing protein |
| 97 | Seita.5G049600 | V | M | 1.0 | 4463951 | 4464657 | peroxiredoxin |
| 98 | Seita.5G049700 | V | M | 1.0 | 4468356 | 4481801 | phenazine biosynthesis protein |
| 99 | Seita.5G050300 | V | H | 1.0 | 4487344 | 4487650 | hypothetical protein |
| 100 | Seita.5G051000 | V | H | 1.0 | 4556919 | 4558227 | cytochrome P450 71D10 |
| 101 | Seita.5G051800 | V | M | 1.0 | 4613981 | 4614084 | mitochondrial carrier protein |
| 102 | Seita.5G052000 | V | H | 1.0 | 4623670 | 4624241 | Pyridine nucleotide-disulphide oxidoreductase family protein |
| 103 | Seita.5G052200 | V | M | 1.0 | 4634752 | 4635040 | CRAL/TRIO domain containing protein |
| 104 | Seita.5G059600 | V | L | 0 | 5282243 | 5293056 | NAK-like ser/thr protein kinase |
| 105 | Seita.5G062900 | V | L | 0 | 5495242 | 5496002 | polyprenyl synthetase |
| 106 | Seita.5G244800 | V | L | 0.42 | 30713985 | 30714207 | receptor-like protein kinase |
| 107 | Seita.5G245200 | V | L | 0.33 | 30735066 | 30736506 | lactate/malate dehydrogenase |
| 108 | Seita.5G245300 | V | L | 0.41 | 30741582 | 30742790 | GDSL-like lipase/acylhydrolase |
| 109 | Seita.5G246500 | V | L | 0.31 | 30827047 | 30831503 | chloroplast unusual positioning protein |
| 110 | Seita.5G247600 | V | L | 0.35 | 30897093 | 30897345 | fruit bromelain precursor |
| 111 | Seita.5G251600 | V | M | 0.58 | 31453411 | 31454136 | glycosyl hydrolase |
| 112 | Seita.5G251700 | V | M | 0.53 | 31468236 | 31468376 | pyruvate kinase |
| 113 | Seita.5G251900 | V | M | 0.71 | 31485698 | 31486085 | Ripening-related family protein precursor |
| 114 | Seita.5G252500 | V | L | 0.44 | 31537377 | 31541586 | F-box and DUF domain containing protein |
| 115 | Seita.5G254100 | V | M | 0.76 | 31625772 | 31628627 | Protein kinase domain containing protein |
| 116 | Seita.5G261000 | V | M | 0.62 | 32368735 | 32370679 | serine/threonine-protein kinase |
| 117 | Seita.6G046300 | VI | L | 0.43 | 3621543 | 3621677 | AP2 domain containing protein |
| 118 | Seita.6G047200 | VI | L | 0.65 | 3679413 | 3679806 | strictosidine synthase |
| 119 | Seita.6G047400 | VI | L | 0.46 | 3705163 | 3706490 | DNA-directed RNA polymerase subunit |
| 120 | Seita.6G047500 | VI | M | 0.62 | 3714955 | 3715941 | Glutaredoxin family protein |
| 121 | Seita.6G047800 | VI | M | 0.57 | 3730239 | 3730511 | vacuolar ATP synthase subunit D 1 |
| 122 | Seita.6G048000 | VI | H | 0.69 | 3734716 | 3741467 | HEAT repeat family protein |
| 123 | Seita.6G049200 | VI | M | 0.53 | 3851267 | 3853235 | plant protein of unknown function domain containing protein |
| 124 | Seita.6G049600 | VI | L | 0 | 3889273 | 3890012 | Protein of unknown function |
| 125 | Seita.7G123100 | VII | L | 0 | 21861875 | 21862161 | aldose 1-epimerase |
| 126 | Seita.7G127700 | VII | L | 0 | 22154396 | 22155646 | O-acyltransferase |
| 127 | Seita.7G132800 | VII | M | 0.48 | 22527886 | 22527887 | C2H2 type zinc finger transcription factor family |
| 128 | Seita.7G133500 | VII | M | 0.55 | 22595883 | 22598206 | glycerophosphoryl diester phosphodiesterase family protein |
| 129 | Seita.7G133800 | VII | M | 0.79 | 22613290 | 22615228 | F-box domain and LRR containing protein |
| 130 | Seita.7G133900 | VII | L | 0.66 | 22624072 | 22624073 | RNA recognition motif containing protein |
| 131 | Seita.7G134300 | VII | L | 0.34 | 22650015 | 22650900 | F-box and DUF domain containing protein |
| 132 | Seita.7G134500 | VII | M | 0.56 | 22667838 | 22670078 | anthranilate phosphoribosyltransferase |
| 133 | Seita.7G135200 | VII | M | 0.70 | 22733640 | 22734427 | beta glucosidase 13 |
| 134 | Seita.7G135600 | VII | M | 0.68 | 22761324 | 22761366 | vacuolar sorting protein 9 domain-containing protein |
| 135 | Seita.7G136200 | VII | M | 0.69 | 22786358 | 22786454 | Carbohydrate-binding X8 domain superfamily protein |
| 136 | Seita.7G136400 | VII | H | 0.89 | 22789949 | 22790431 | Protease inhibitor/seed storage/LTP family protein precursor |
| 137 | Seita.7G136800 | VII | M | 0.73 | 22833191 | 22836394 | GRAM and C2 domains containing protein |
| 138 | Seita.7G137700 | VII | M | 0.74 | 22892559 | 22892559 | NAC (No Apical Meristem) domain transcriptional regulator superfamily protein |
| 139 | Seita.7G138300 | VII | M | 0.58 | 22931446 | 22936235 | purine permease |
| 140 | Seita.7G139300 | VII | L | -0.63 | 23020540 | 23020723 | cis-zeatin O-glucosyltransferase |
| 141 | Seita.7G140000 | VII | H | 0.85 | 23058064 | 23060452 | protein-L-isoaspartate O-methyltransferase |
| 142 | Seita.8G093500 | VIII | L | -0.76 | 11933823 | 11935298 | expressed protein |
| 143 | Seita.8G094000 | VIII | L | -0.67 | 11985326 | 11985522 | NBS-LRR disease resistance protein |
| 144 | Seita.8G094300 | VIII | L | -0.60 | 12014436 | 12014960 | Putative Clp protease homologue |
| 145 | Seita.8G094400 | VIII | L | -0.77 | 12015738 | 12017814 | C2H2 zinc finger protein |
| 146 | Seita.8G094700 | VIII | L | -0.79 | 12073873 | 12076289 | bZIP transcription factor domain containing protein |
| 147 | Seita.8G096400 | VIII | L | -0.69 | 12546488 | 12547097 | 1-aminocyclopropane-1-carboxylate oxidase homolog 1 |
| 148 | Seita.8G097000 | VIII | L | -0.83 | 12640978 | 12694073 | serine carboxypeptidase-like 42 |
| 149 | Seita.8G097300 | VIII | L | 0 | 12701952 | 12707653 | phenylalanyl-tRNA synthetase beta chain |
| 150 | Seita.8G101700 | VIII | L | -0.76 | 13591482 | 13593591 | stripe rust resistance protein Yr10 |
| 151 | Seita.8G104000 | VIII | L | -0.64 | 13962214 | 13962763 | The CR4L subfamily has homology with Crinkly4 |
| 152 | Seita.8G104700 | VIII | L | -0.63 | 14114677 | 14115433 | xylem cysteine peptidase 1 |
| 153 | Seita.8G106200 | VIII | L | -0.73 | 14504281 | 14597052 | glutathione peroxidase |
| 154 | Seita.8G111200 | VIII | L | -0.61 | 18483619 | 18492727 | AMP-binding domain containing protein |
| 155 | Seita.8G111500 | VIII | L | -0.68 | 18682472 | 18685324 | peptide transporter PTR2 |
| 156 | Seita.8G114400 | VIII | L | -0.81 | 20258562 | 20260500 | retrotransposon protein |
| 157 | Seita.8G122100 | VIII | L | -0.73 | 23159570 | 23161455 | oxidoreductase, 2OG-Fe oxygenase family protein |
| 158 | Seita.8G123400 | VIII | L | -0.88 | 23541408 | 23541900 | plastocyanin-like domain containing protein |
| 159 | Seita.8G132800 | VIII | L | -0.78 | 25649248 | 25652291 | Leucine Rich Repeat family protein |
| 160 | Seita.8G135400 | VIII | L | -1.0 | 26216186 | 26220388 | F-box and other domain containing protein |
| 161 | Seita.8G135600 | VIII | L | -0.56 | 26249206 | 26249574 | inducer of CBF expression 1 |
| 162 | Seita.8G142600 | VIII | L | -0.67 | 27705249 | 27706679 | plant protein of unknown function domain containing protein |
| 163 | Seita.8G144900 | VIII | L | -0.81 | 28039251 | 28041065 | aspartyl/glutamyl-tRNA amidotransferase subunit B |
| 164 | Seita.8G145000 | VIII | L | -0.69 | 28044317 | 28051526 | centromere/kinetochore protein zw10 |
| 165 | Seita.9G043500 | IX | L | -0.50 | 2447678 | 2447954 | cell division control protein 2 homolog 3 |
| 166 | Seita.9G043600 | IX | L | -0.48 | 2454749 | 2455840 | ubiquitin family protein |

Note: Snp effect sizes (Snp Eff), High (H), Moderate (M), Low (L).

Table S6. Quality statistics of mapping with the reference genome for RNA-Seq.

| **Samples** | **Raw-reads** | **Clean-reads** | **Clean bases (G)** | **Total-map** | **Q30** | **GC ratio (%)** |
| --- | --- | --- | --- | --- | --- | --- |
| CK_Yu28_1 | 49,723,490 | 47,867,765 | 6.19 | 96.27% | 93.86% | 46.6 |
| CK_Yu28_2 | 56,071,668 | 51,025,891 | 6.24 | 91.00% | 93.98% | 46.88 |
| CK_Yu28_3 | 47,479,344 | 45,795,059 | 6.21 | 96.45% | 94.00% | 46.74 |
| LN_Yu28_1 | 51,517,318 | 49,627,859 | 6.17 | 96.33% | 94.06% | 47.72 |
| LN_Yu28_2 | 47,885,170 | 45,901,207 | 6.30 | 95.86%) | 93.90% | 47.15 |
| LN_Yu28_3 | 44,286,606 | 42,490,997 | 6.25 | 95.95% | 93.67% | 46.96 |
| CK_Qiyehuang_1 | 46,859,399 | 44,654,514 | 6.14 | 94.87% | 93.68% | 46.75 |
| CK_Qiyehuang_2 | 45,872,309 | 43,657,711 | 6.33 | 95.76% | 93.79% | 47.36 |
| CK_Qiyehuang_3 | 47,567,603 | 45,369,512 | 6.20 | 96.27% | 94.50% | 47.38 |
| LN_Qiyehuang_1 | 50,719,871 | 48,639,473 | 6.15 | 94.85% | 93.49% | 47.24 |
| LN_Qiyehuang_2 | 48,346,597 | 46,549,621 | 6.31 | 95.64% | 93.37% | 47.54 |
| LN_Qiyehuang_3 | 47,864,367 | 45,357,752 | 6.26 | 94.34% | 93.36% | 47.38 |


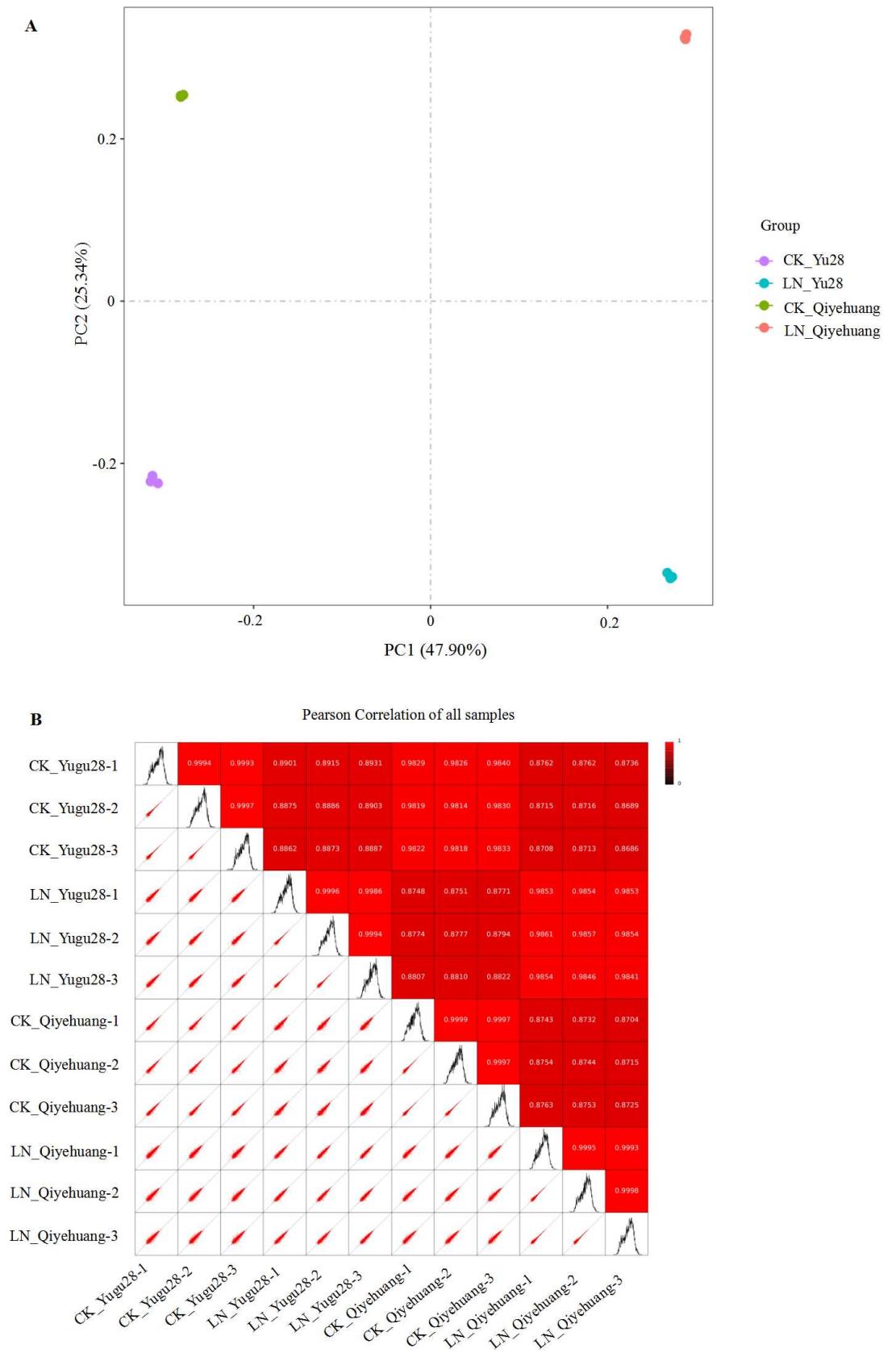


**Fig. S1** Quality Control of RNA-seq Data. (A) PCA plot (PC1 vs PC2), (B) Sample-to-sample correlation heatmap.

**
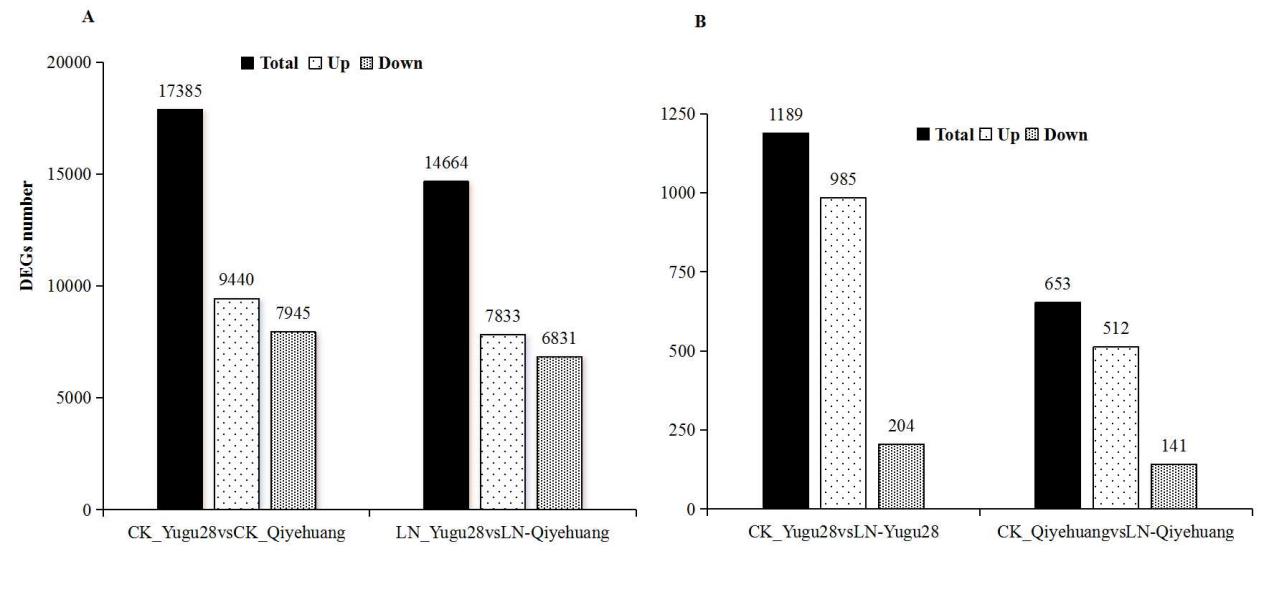
Fig. S2** Differences in gene expression in the leaves of two foxtail millet genotypes (Yugu28, Qiyehuang) between the genotypes under CK and LN. The number of differentially expressed genes (DEGs) between Yugu28 and Qiyehuang (A) in the CK and LN treatments and between the CK and LN treatments (B) of Yugu28 and Qiyehuang.


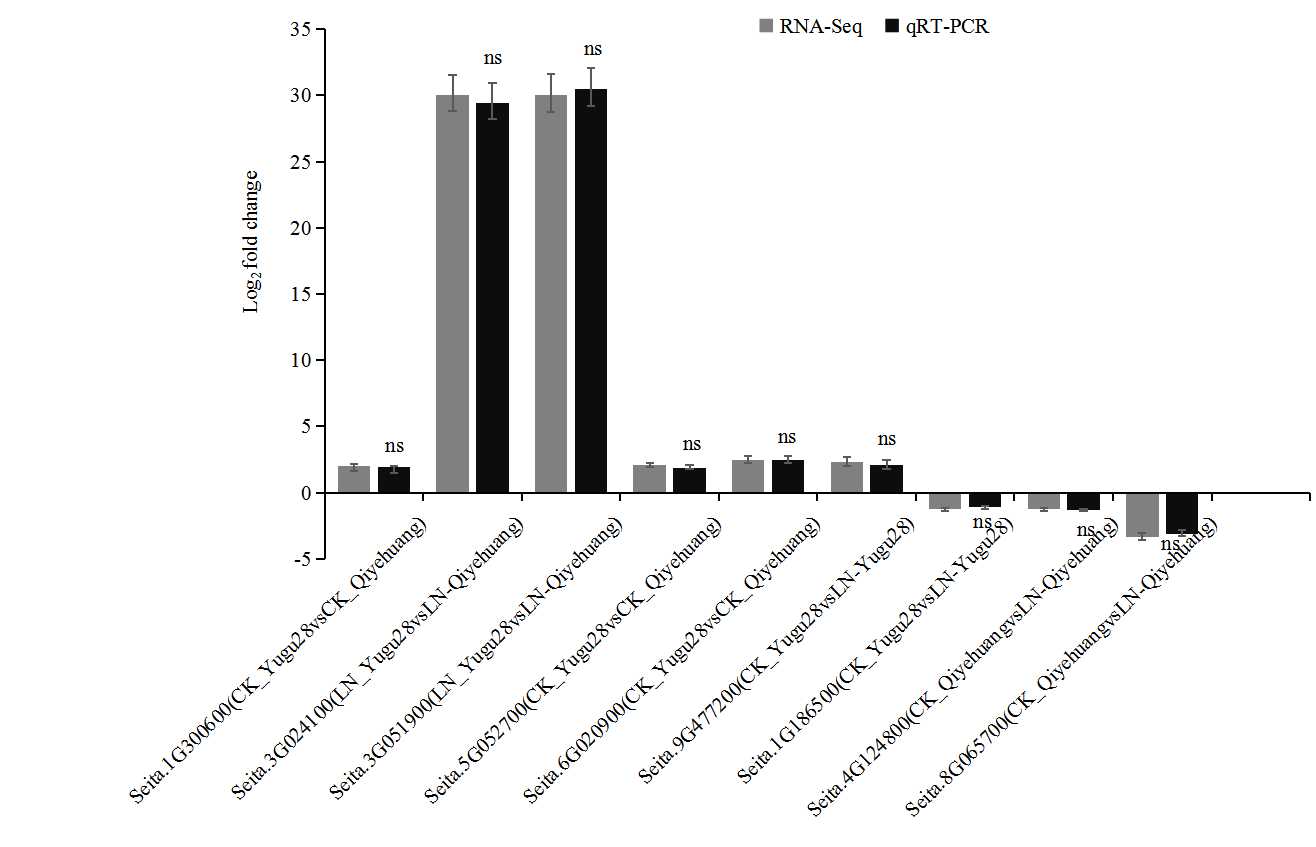
**Fig. S3** Confirmation of transcript levels of differentially expressed genes (DEGs) between Yugu28 and Qiyehuang. Transcript levels of selected DEGs were analyzed by qRT-PCR and RNA-seq. Data are presented as mean ± SD (n=3). Asterisks indicate statistically significant differences (ns, not statistically significant) according to Student’s *t-test*.

**
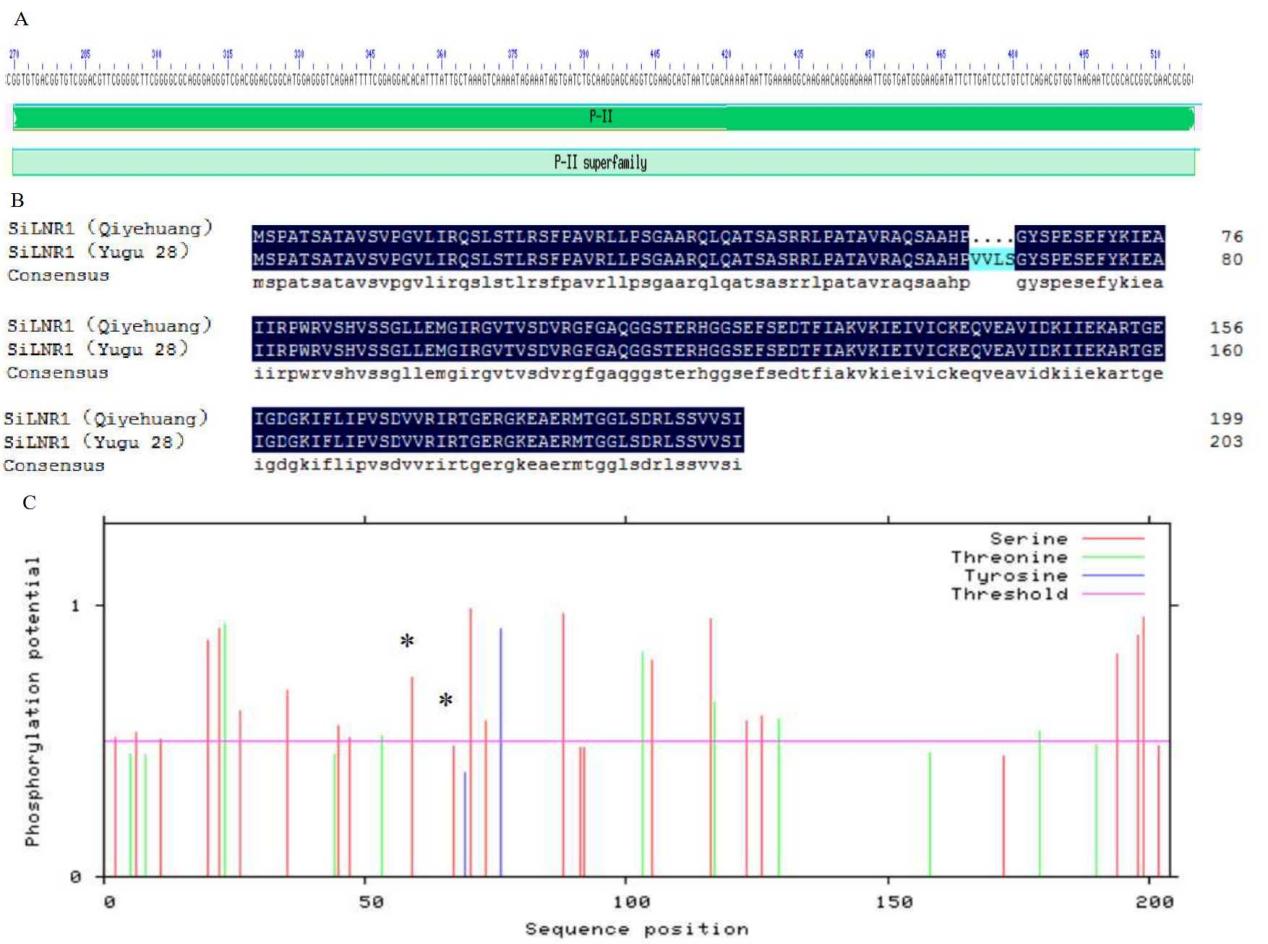
**

**Fig. S4** Comparison of *SiLNR1* protein sequence of the LN-tolerant foxtail millet (Yugu28) and the LN-sensitive foxtail nillet (Qiyehuang), and prediction of the phosphorylation site. Conservative domain analysis of *SiLNR1* (A). Protein sequence alignment of *SiLNR1* between LN-tolerant foxtail millet (Yugu28) and the LN-sensitive foxtail millet (Qiyehuang) (B). Phosphorylation site prediction of the *SiLNR1* protein; The asterisks represent the mutation of two amino acids of *SiLNR1* in Qiyehuang to phosphorylation sites in Yugu28 (C).


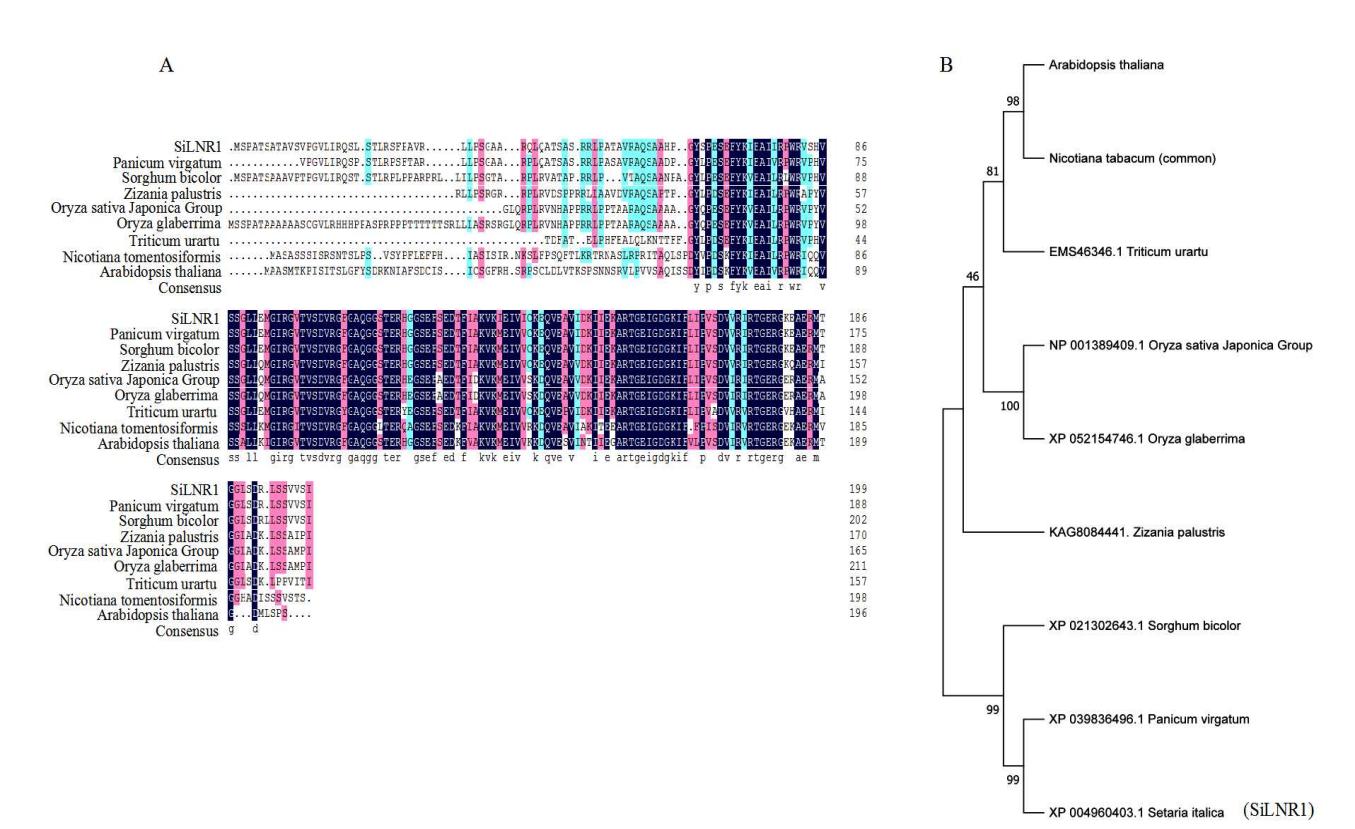
**Fig. S5** Comparison and evolutionary relationship analysis of *SiLNR1* amino acid sequences among different species. Amino acid sequences alignment of *SiLNR1* in *Panicum virgatum*, *Sorghum bicolor*, *Zizania palustris*, *Oryza sativa Japonica Group*, *Oryza glaberrima*, *Triticum urartu*, *Nicotiana tomentosiformis*, *Arabidopsis thaliana*(A). Phylogenetic tree construction of *SiLNR1* in *Panicum virgatum*, *Sorghum bicolor*, *Zizania palustris*, *Oryza sativa Japonica Group*, *Oryza glaberrima*, *Triticum urartu*, *Nicotiana tomentosiformis*, *Arabidopsis thaliana* (B).

**
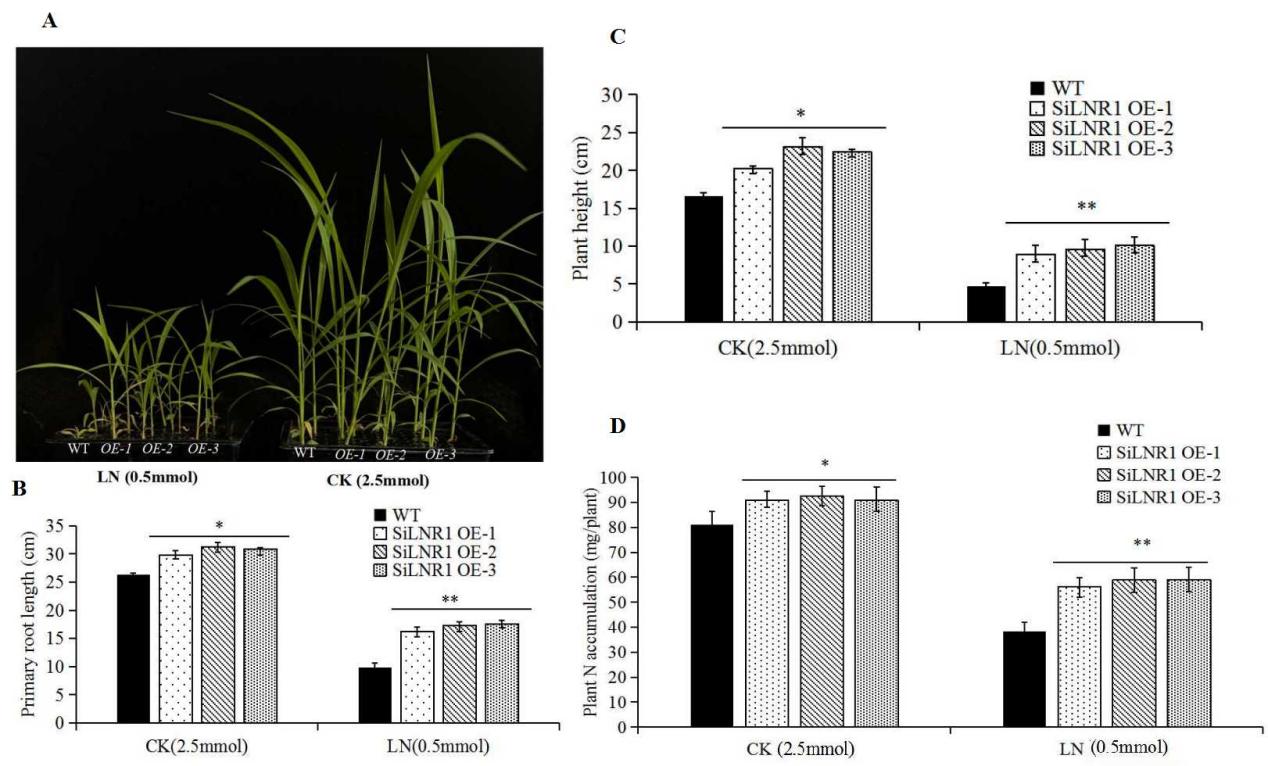
**

**Fig. S6** Phenotypic identification of *SiLNR1*-overexpressing plants. Phenotypes of the wild-type and *SiLNR1*-overexpressing plants under CK and LN conditions (A); primary root length (B); plant height (C); plant nitrogen accumulation (D). Data are presented as mean ± SD (n=6 biologically independent plants). Asterisks indicate statistically significant differences (** *P* < 0.01, * *P* < 0.05) according to Student’s *t-test*.

**
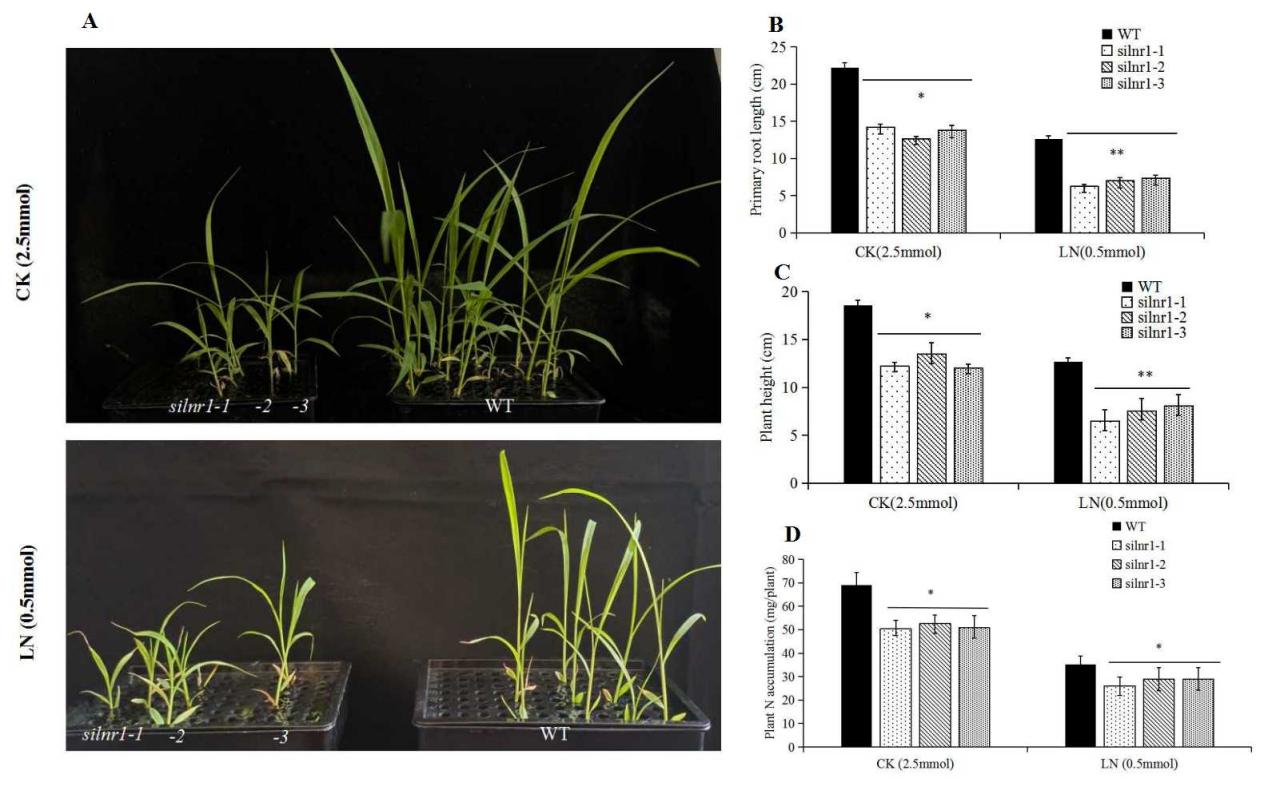
**

**Fig. S7** Phenotypic identification of *silnr1* mutants. Phenotypes of the wild-type and *silnr1* mutants under CK and LN conditions (A); primary root length (B); plant height (C); plant nitrogen accumulation (D). Data are presented as mean ± SD (n = 6 biologically independent plants). Asterisks indicate statistically significant differences (** *P* < 0.01, * *P* < 0.05) according to Student’s *t-test*.
