# Supplementary figures and images for: SiLNR1-Mediated Nitrogen Regulatory Signaling Enhances Nitrogen Use Efficiency and Grain Yield in Foxtail Millet (Setaria italica L.) under Low-Nitrogen Stress
Source: Research (Wash D C). 2026 Feb 25;9:1148. doi: 10.34133/research.1148 (PMC12932939; doi:10.34133/research.1148)

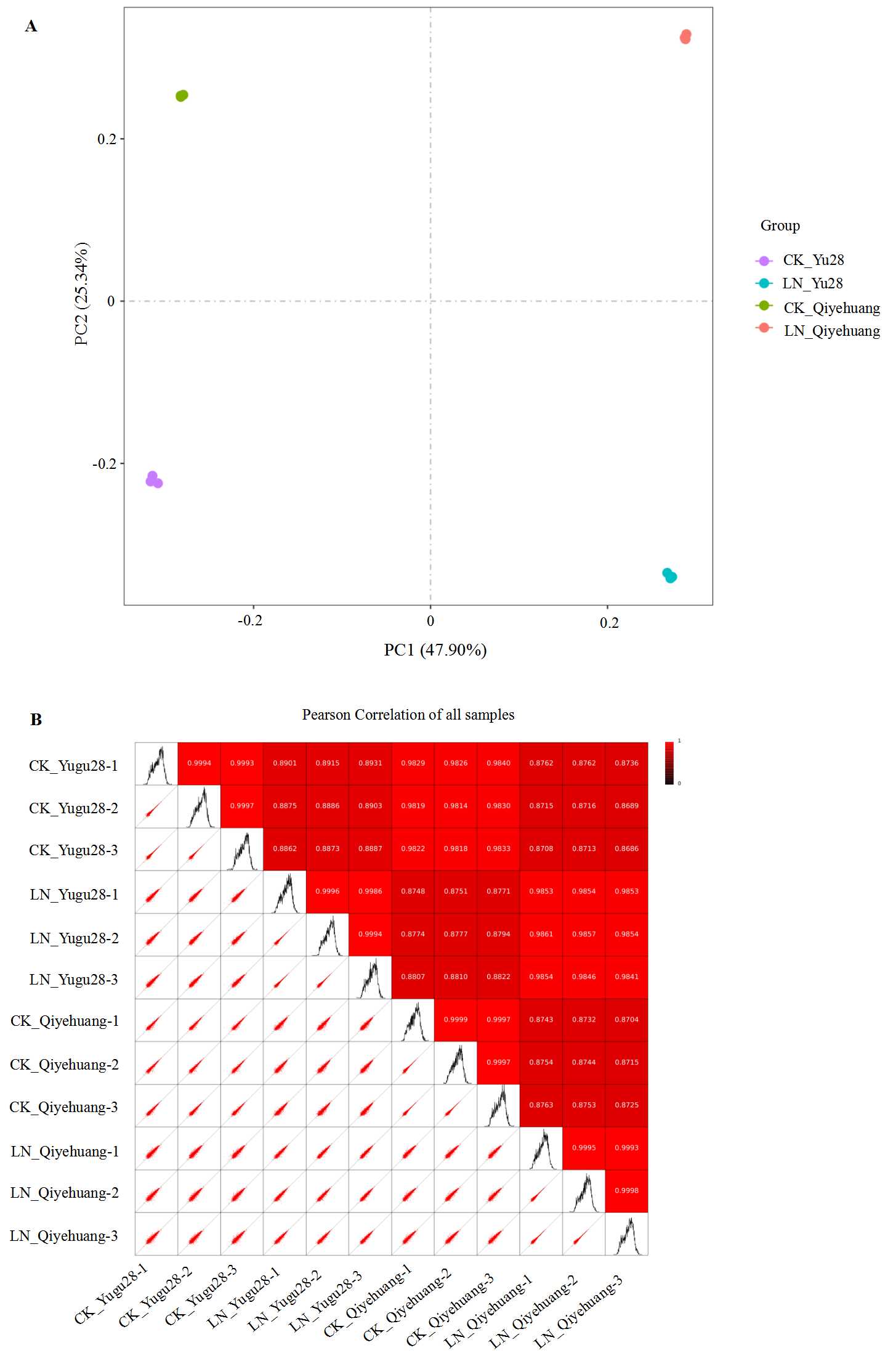

Supplement: Supplementary 1 — Figs. S1 to S7 Tables S1 to S6 [file research.1148.f1.zip › Figure S1.jpg]

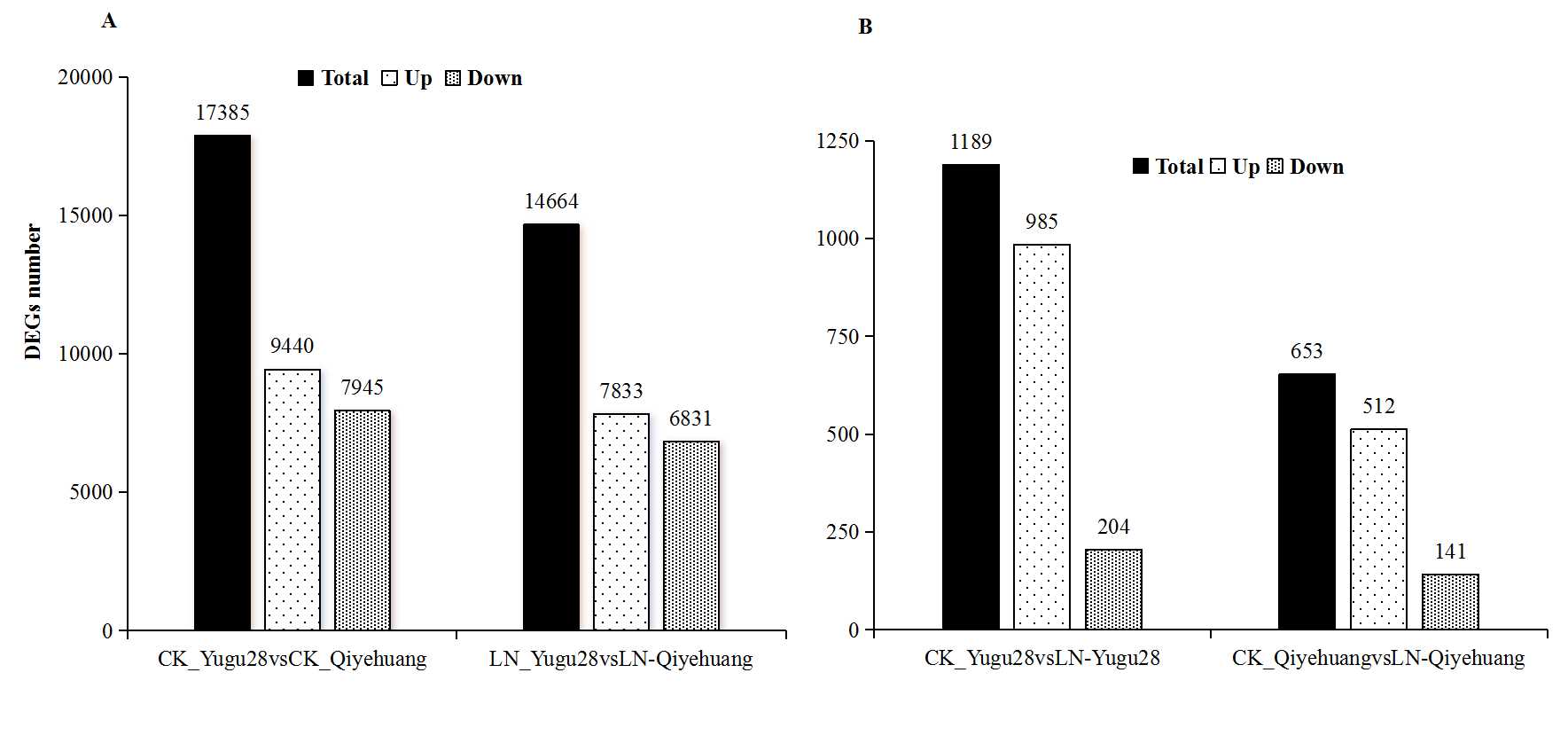

Supplement: Supplementary 1 — Figs. S1 to S7 Tables S1 to S6 [file research.1148.f1.zip › Figure S2.jpg]

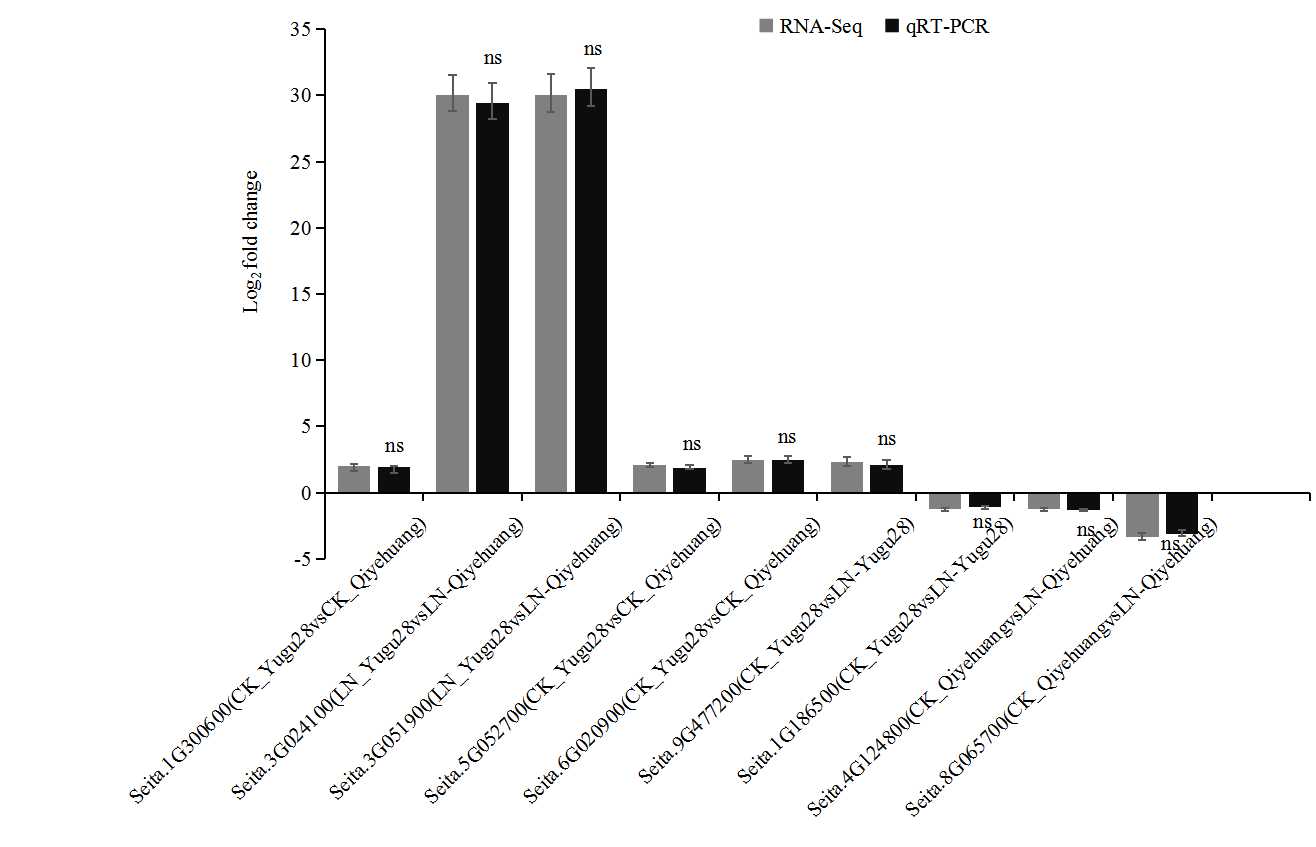

Supplement: Supplementary 1 — Figs. S1 to S7 Tables S1 to S6 [file research.1148.f1.zip › Figure S3.jpg]

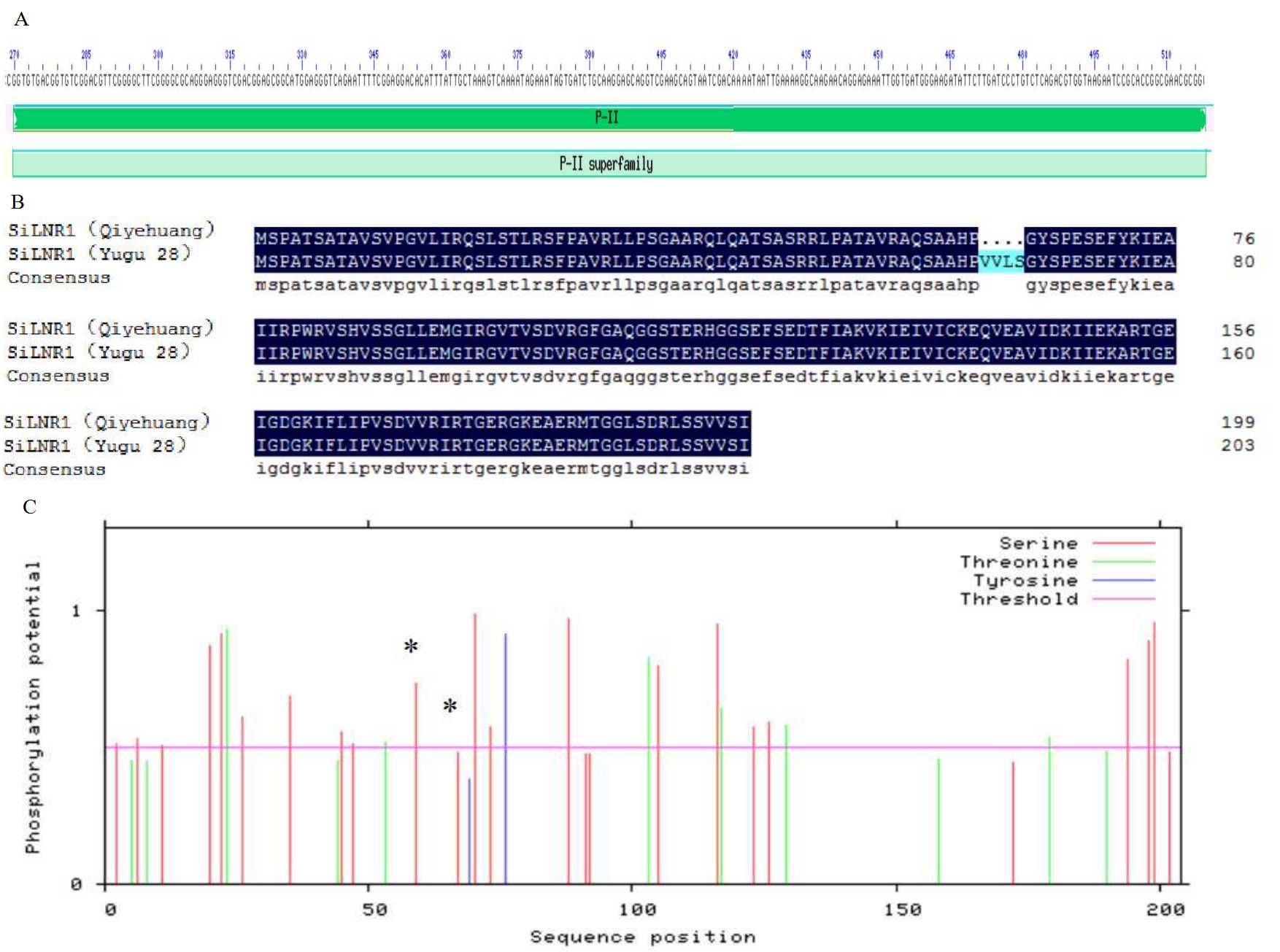

Supplement: Supplementary 1 — Figs. S1 to S7 Tables S1 to S6 [file research.1148.f1.zip › Figure S4.jpg]

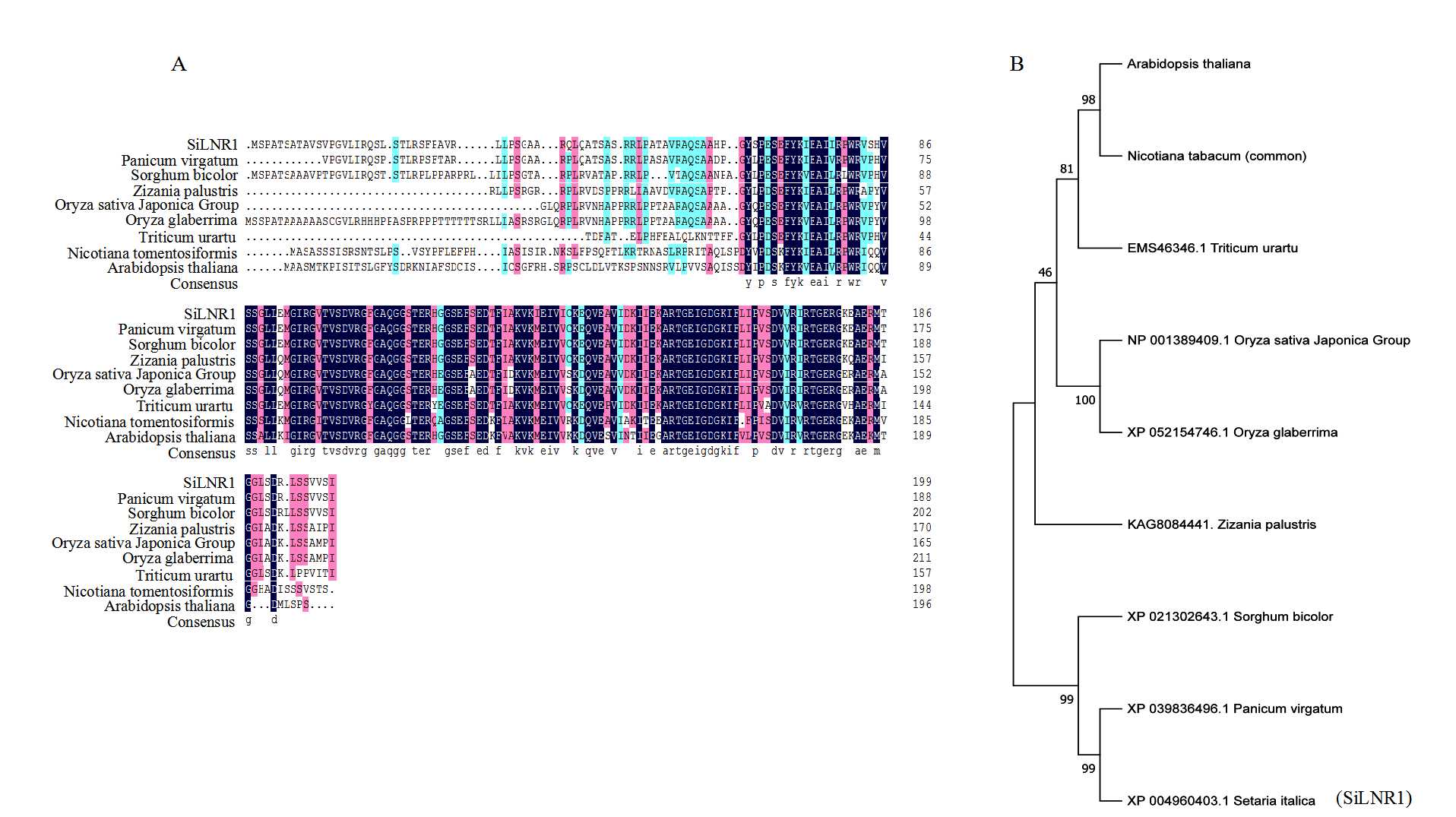

Supplement: Supplementary 1 — Figs. S1 to S7 Tables S1 to S6 [file research.1148.f1.zip › Figure. S5.jpg]

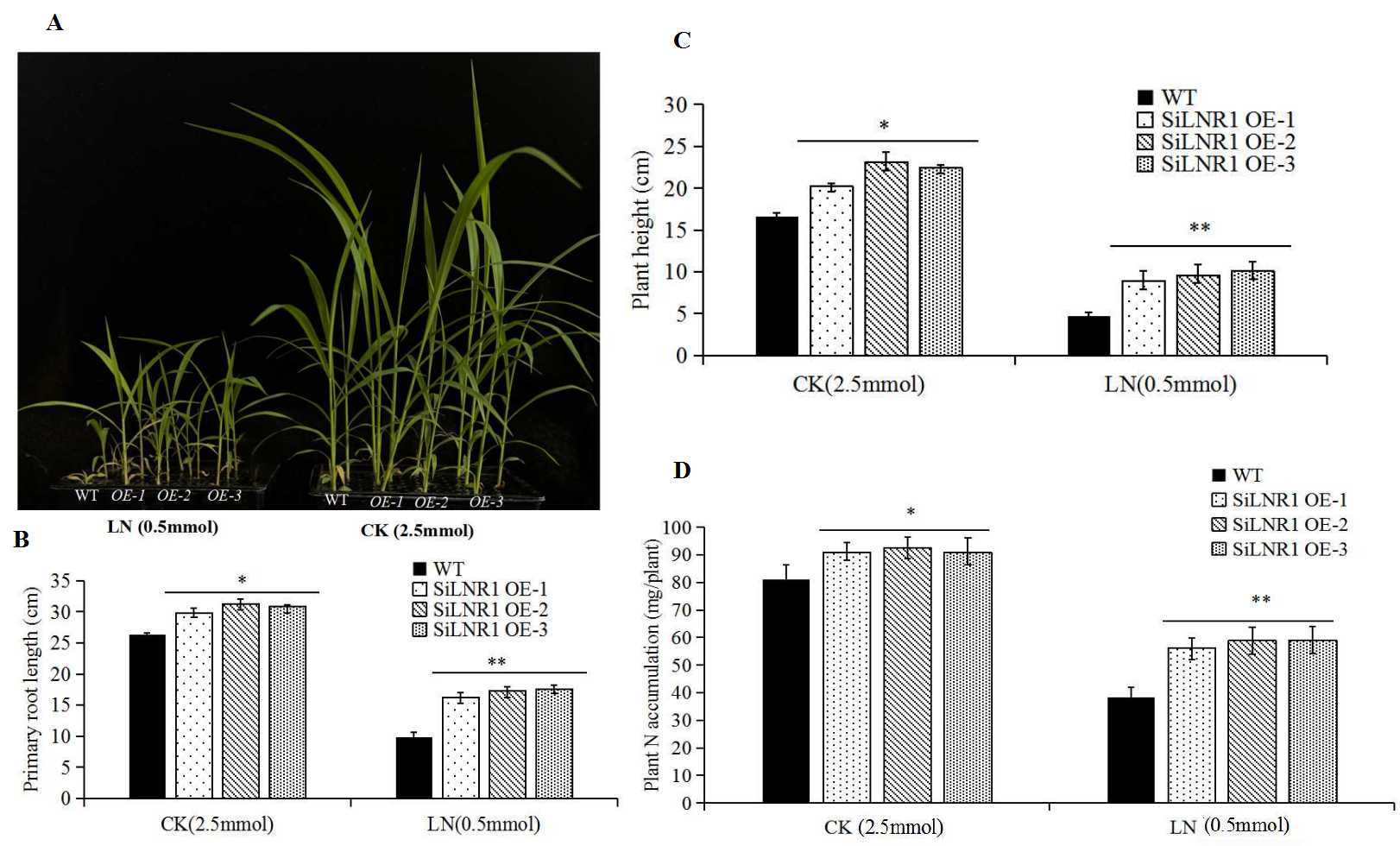

Supplement: Supplementary 1 — Figs. S1 to S7 Tables S1 to S6 [file research.1148.f1.zip › Figure. S6.jpg]

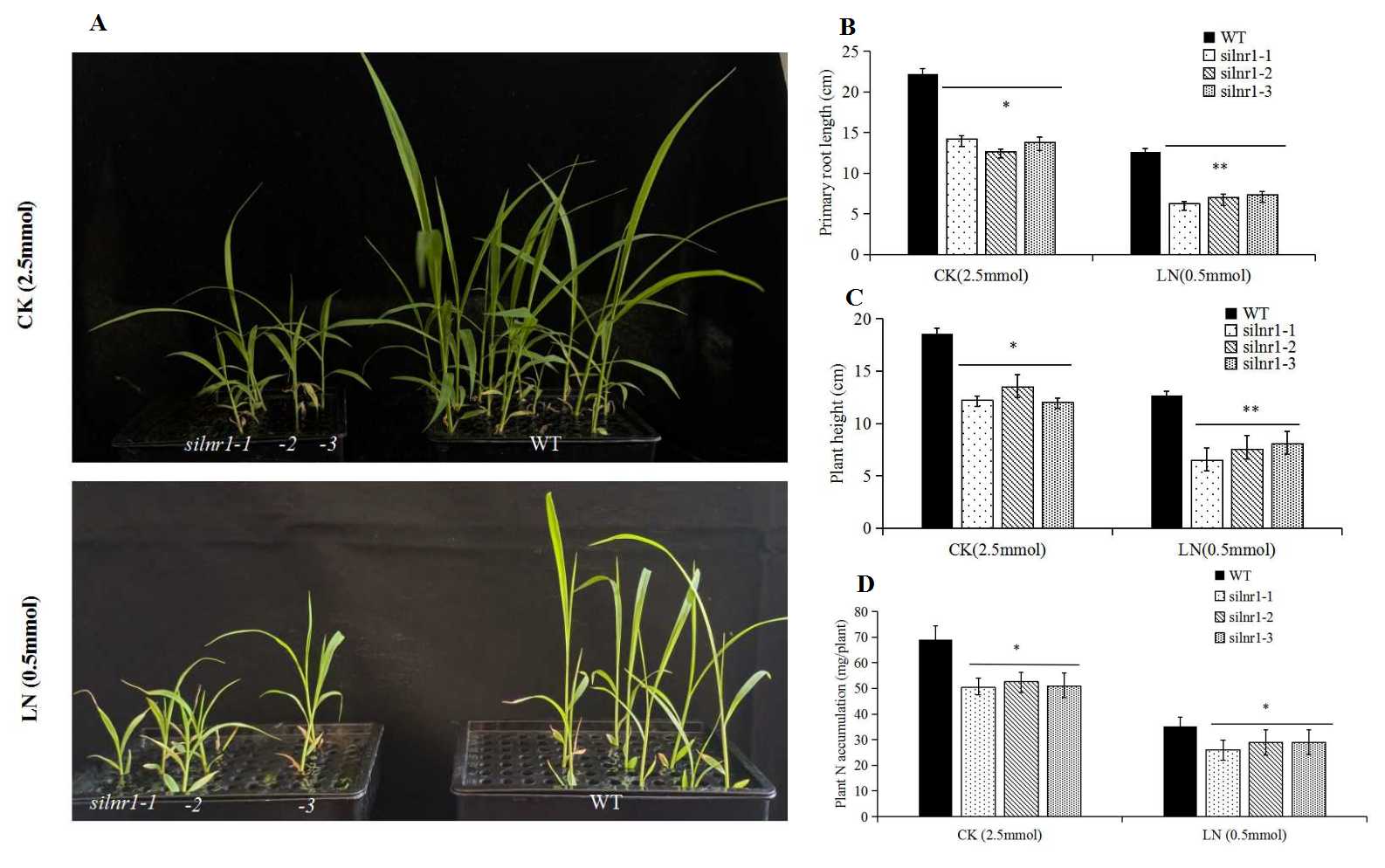

Supplement: Supplementary 1 — Figs. S1 to S7 Tables S1 to S6 [file research.1148.f1.zip › Figure. S7.jpg]
